# *Ab initio* study on photocatalytic properties of PtSSe–WXY Janus heterostructures

**DOI:** 10.1039/d6ra03413f

**Published:** 2026-07-03

**Authors:** Shivprasad S. Shastri, Antonio Cammarata, Tomas Polcar

**Affiliations:** a Department of Control Engineering, Faculty of Electrical Engineering, Czech Technical University in Prague Karlovo Náměstí 13 Prague Czech Republic cammaant@fel.cvut.cz shastshi@fel.cvut.cz +420 224 91 8646 +420 224 35 5713; b Engineering Materials & nCATS, FEE, University of Southampton SO17 1BJ Southampton UK

## Abstract

Semiconductor photocatalysis offers a sustainable route for converting solar energy into chemical energy, enabling the production of clean fuels and valuable chemical products. To this aim, we explore van der Waals heterostructures made up of Janus PtSSe and WXY (X, Y = S, Se, Te and X ≠ Y), in the context of photocatalytic applications. The redox capabilities of various heterostructure configurations (atom facing types and stacking orders) are evaluated by aligning the absolute band edge positions with respect to redox potentials of hydrogen and oxygen evolution reaction (HER and OER) and CO_2_ reduction reactions. The stability of photocatalyst candidates is checked by layer binding energy, elastic constant calculations and *ab initio* molecular dynamics simulations. The optical absorption spectra suggest good light absorption in the visible range. Further, strain engineering is applied as a way to tune band edges and evaluate the possible use of the heterostructures as photocatalysts. This study shows that van der Waals heterostructure bilayers composed of Janus PtSSe and WSeTe in specific geometric configurations can be potential materials as photocatalysts for HER, OER and CO_2_ reduction reactions. Finally, we suggest that, although systems made up of PtSSe and WSTe cannot be used for photocatalytic applications, they can be explored for applications in thermoelectric energy conversion or infrared photovoltaics.

## Abstract 2

van der Waals heterostructure bilayers composed of Janus PtSSe and WSeTe in specific geometric configurations can be potential materials as photocatalysts for HER, OER and CO_2_ reduction reactions.

## Graphical abstract

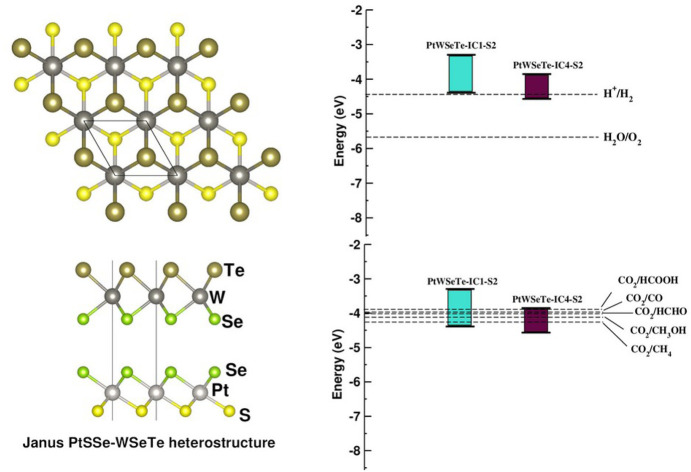

## 1 Introduction

The conversion of solar energy into chemical energy through photocatalysis provides an alternative pathway for generating clean, renewable, and sustainable fuels and useful chemical products.^1–4^ Photocatalytic reduction of CO_2_ into useful chemicals such as alkanes, alcohols and aldehydes is one of the viable strategies to reduce CO_2_ emissions or capture it from the environment.^5,6^ Generation of hydrogen and oxygen gases using photocatalytic materials by splitting water promises an environment friendly, clean and renewable energy source.^6–8^ To this aim, development of such photocatalytic materials with tunable electronic properties, optimized for light absorption and band edge positions suitable for driving these desired chemical reactions, is crucial.^9^

Many materials have been explored for photocatalytic water splitting to produce hydrogen and oxygen, including 2D materials.^10–17^ Similarly, a number of bulk and 2D materials are studied for photocatalytic reduction of CO_2_.^5,18–20^ 2D materials have been actively studied for their use in catalysis, photovoltaics, battery electrodes, electronics, optoelectronics and their interesting new physics.^21–24^ Relative to bulk materials, 2D materials are characterised by large surface area for photon absorption, enhanced charge separation, structural flexibility, cocatalyst integration and higher surface-water interaction.^7,25–27^ These features are present thanks to their reduced thickness which shortens carrier migration distances to the surface, then reducing recombination possibility and providing more carriers for reactions.^7,17^ In 2D monolayer semiconductors both electron and hole generation needed for redox reactions takes place in the same surface, resulting in possible high recombination of carriers.^26,28^ Arranging different 2D semiconductor materials in stackings to create heterostructures (HS) can be a promising strategy to design and tailor materials for specific properties, functionalities and to overcome the drawback of high carrier recombinations.^17,29–31^ These HSs can show type-I (straddled), type-II (staggered) or type-III (disjoint) band alignments.^17^ The type-I HS can be used for photocatalysis, although they can be less effective because of limited ability to separate the photogenerated charge carriers; instead, type-II band alignment facilitates electrons and holes generation separately in two distinct stacked layers, reducing carrier recombination and showing enhanced performance.^17,31–33^ Density functional theory (DFT) based *ab initio* calculations are useful to design such new HSs, predicting their properties and feasibility of synthesis thereby supporting experimental realisation of new materials and explaining the experimentally observed properties.^34–38^

2D materials such as transition metal monosulfides, graphitic carbon nitrides, transition metal dichalcogenides (TMDC), ZnO, Blue P and their HSs are currently studied for photocatalytic applications.^17,31,39^ In particular, the Janus type of TMDCs have attracted interest due to inherent electric field and dipole moment arising from the out of plane mirror symmetry breaking.^40,41^ In contrast to TMDCs, the intrinsic dipole moment in Janus TMDCs has been reported to help in photo-induced electron–hole separation and elongating carrier recombination time, an important feature to collect charges and improve efficiency in photocatalytic materials.^40–42^ Experimentally synthesised 2D Janus MoSSe is reported to show HER activity which is supported by DFT calculations.^43^ Similarly, monolayer of Janus MoSSe is studied by *ab initio* calculations and found to be a potential water splitting photocatalyst.^44^ The monolayer and bilayers of MoXY and WXY (X, Y = S, Se, Te) are studied by C. Xia *et al.* by ab inito calculations which suggested these materials are promising candidates for photocatalysis with internal electric field facilitating carrier separation.^45^ Another study on Janus MoXY by Y. Ji *et al.* (X, Y = O, S, Se, Te) investigated their photocatalytic activity by band edge alignment which showed favourable band edge position relative to water redox potential.^46^ Energetically and dynamically stable monolayers of new Janus monolayers TiSO, ZrSO and HfSO are also reported as new promising candidates for photocatalytic water splitting as shown in ref. 47. Study on Tungsten based 2D Janus WSSe monolayer shows that it possesses suitable band edge position along with strong optical absorbance and high carrier separation which can be well modulated by the application of strain.^48^ Similarly, monolayers of PtXY (X, Y = S, Se, Te) are also studied as candidates for photocatalytic water splitting. These studies show that they are potential candidates with in-build electric field and predicted theoretical feasibility of synthesis.^49,50^ Heterostructure bilayers offer further advantage as photocatalysts to tune the properties as discussed earlier. Such HS bilayers *viz.* Janus MoSSe with ZnO,^3^ bilayer of MoSSe,^51^ MoXY with triazine based graphitic carbon nitride (TGCN)^52^ and HSs of MX_2_ (M = Mo, W; X = S, Se)–MXY (M = Mo, W; X, Y = S, Se, Te)^32^ are explored for photocatalytic redox reactions of water. However, the HSs made up of PtSSe and WXY monolayers have not yet been studied for photocatalytic water splitting or CO_2_ reduction reactions. Creating HS bilayers from these two Janus monolayers which show photocatalytic activity can be promising to achieve carrier generation in separate layers and overcome high carrier recombination. This motivates the present study, which aims to contribute to the discovery of new Janus heterostructures for photocatalysis applications.

In this work, we consider TMDC-based Janus heterostructures, namely 1T-PtSSe/2H-WSSe (PtWSSe), 1T-PtSSe/2H-WSTe (PtWSTe) and 1T-PtSSe/2H-WSeTe (PtWSeTe), and study the stability, electronic and optical properties at various interface configurations and stacking orders. By checking suitable alignment of the band edge positions with respect to vacuum level and comparing with oxidation–reduction potentials, we suggest that these materials are good candidates for hydrogen evolution reaction (HER), oxygen evolution reaction (OER) and CO_2_ reduction applications.

## 2 Computational methods

**Fig. 1:**
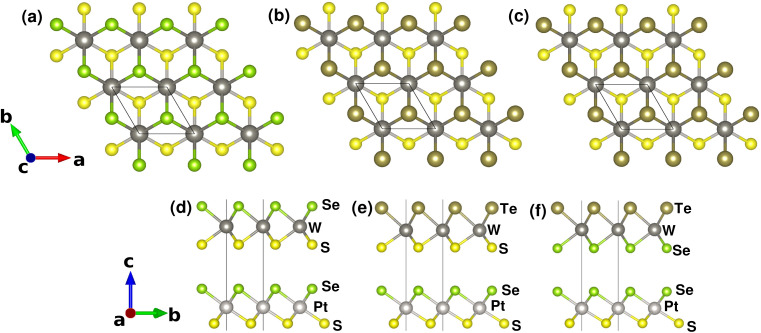
Model geometry of three HSs considered in this work: (a) 1T-PtSSe/2H-WSSe (b) 1T-PtSSe/2H-WSTe and (c) 1T-PtSSe/2H-WSeTe (top view). (d)–(f) Show the respective side views.

We choose 1T-PtSSe and 2H-WXY (X, Y = S, Se, Te and X ≠ Y) 2D monolayers to create bilayer heterostructures; in the model names, “T” and “H” indicate the trigonal and hexagonal lattices, respectively. Previous *ab initio* studies show that these monolayers display photocatalytic HER, OER and water splitting properties.^48–50,53^ The reported cohesive energy values for these monolayers suggests that their formation is energetically favoured,^45,50^ while related phonon spectra support their dynamical stability.^45,49,50^ Thus, we consider these monolayers to build three vertically stacked bilayer HSs in the following way. In our geometric models, we position the monolayers parallel to the crystallographic (*a*, *b*) plane; we then stack the 1T-PtSSe monolayer as the lower layer and 2H-WXY as the upper layer along the *c* crystallographic axis orthogonal to the monolayers plane. The initial in-plane lattice parameters are chosen to be the average of the optimized lattice parameters of the parent monolayers. We then perform full (both lattice and atom position) structure optimizations to get optimized lattice constants and atomic positions for three HSs; the relaxed geometries used in this work together with a graphic representation and related discussion are reported in the sections “structural properties” and “optimised structure files” of the SI. The top and side views of these model structures are shown in Fig. 1.

**Fig. 2:**
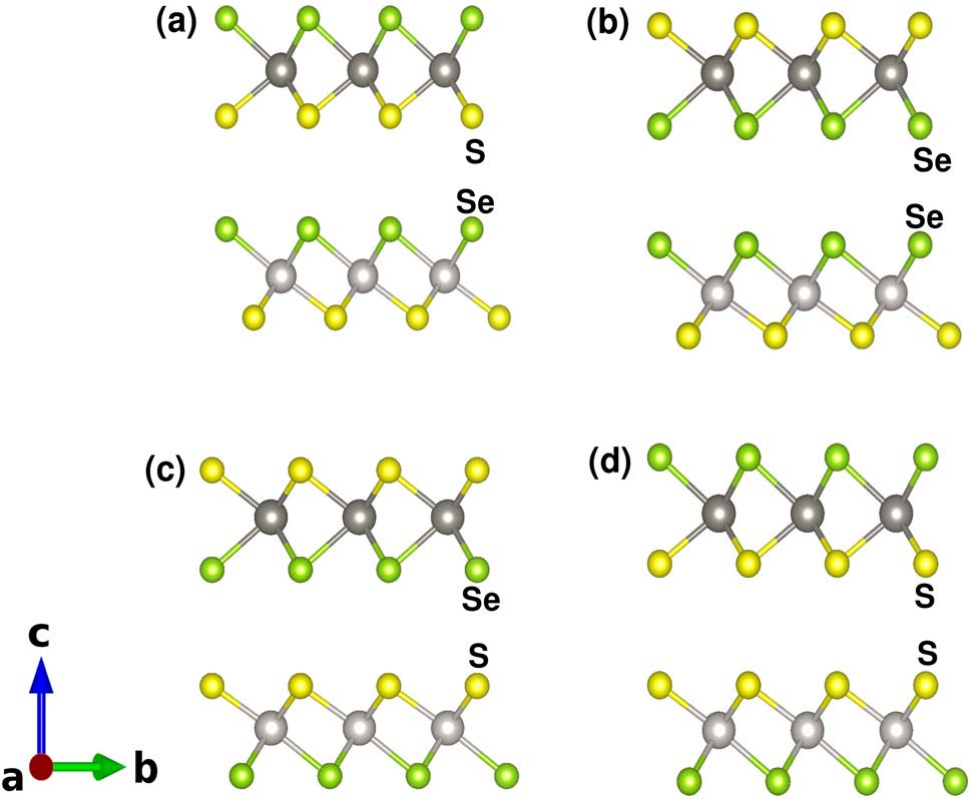
The four possible atom facing configurations, IC1–IC4 ((a)–(d)) in the case of 1T-PtSSe/2H-WSSe HS. Similarly, four possible ICs are considered in case of other two HSs.

Since the parent Janus monolayers shows a lack of inversion symmetry, different atom-facing combinations can be considered at the heterostructure interface. The type of atoms facings at the interface can lead to changes in the electronic structure and band gap due to the overlap of different atom pairs, then acting as a possible way to tune the electronic properties. For a given HS, four atom facing configurations (or interface configurations) are possible at the interface *viz.* S–X, S–Y, Se–X, and Se–Y for a fixed X and Y atom types of the top layer (WXY layer); we will refer to them as IC1–IC4. An example of these four ICs (IC1–IC4) in the case of the 1T-PtSSe/2H-WSSe system is shown in Fig. 2. Similarly, four ICs are considered in the case of 1T-PtSSe/2H-WSTe and 1T-PtSSe/2H-WSeTe HSs. In the construction of a bilayer, different stacking orders are possible depending on the relative atomic positions of the top and bottom monolayers. We consider five stacking orders which are denoted as S1–S5 in what follows; related discussion is given in section “structural properties” of the SI, together with the five different stacking orders. Both the types of interface configurations and stacking orders can affect the electronic structure and might serve to tune the band gap of the overall system.

We perform *ab initio* calculations within the DFT framework with the projector augmented wave method as implemented in the VASP software.^54,55^ Structural relaxation (atomic position and lattice parameters) and *ab initio* molecular dynamics (AIMD) simulations are carried out using Perdew–Burke–Ernzerhof (PBE) energy functional;^56^ the DFT-D3 functional of Grimme *et al.*^57^ is used to account for van der Waals interactions. This choice of energy functional and van der Waals correction is motivated by their use for similar van der Waals materials.^58–62^ The value of the lattice constant *c* is set to 30 Å to avoid interactions between the images along the same direction. The plane wave basis set is truncated with an energy cutoff equal to 500 eV, while the Brillouin zone is sampled using a 13 × 13 × 1 Monkhorst–Pack mesh.^63^ The self-consistent field and force convergence criteria are set to 10^−9^ eV and 10^−4^ eV Å^−1^, respectively. We perform hybrid DFT calculations to get more accurate band gap description using the Heyd–Scuseria–Ernzerhof (HSE06) functional.^64^ These calculations are performed for those HS configurations which showed band gap more than 0.5 eV from preliminary PBE functional estimations, to exclude candidates with narrow band gaps. The HSE06 functional is known to give a good agreement with experimental band gap for conventional 2D TMDCs and in the prediction of band gap for many semiconductors compared to GGA.^65–67^ Therefore, we use the HSE06 hybrid functional to predict band gap values in this work. The thermal stability of heterobilayers is checked by AIMD simulations at 300 K using the NVT ensemble and Nosé–Hoover thermostat, using supercells with dimension 3 × 3 × 1 and a time step of 1 fs for a total of 8 ps (8000 time steps). The frequency dependent dielectric function are obtained using the method of Gajdoš *et al.* as implemented in VASP.^68^ We post-process the data with the VASPKIT utility to obtain optical absorption spectra, layer projected band structures, second order elastic constants and planar averaged electrostatic potentials.^69^

## 3 Results and discussion

### 3.1 Structural properties and stability

The optimized lattice constants and the interlayer distances *d* for stacking order S1 for all the ICs are reported in Table 1, where *d* is the distance between the layers of chalcogen atoms at the interface of the heterostructure. Analogous tables are reported in the SI for the stacking orders S2–S5 (Tables S1–S4). By inspecting the table, we observe that changing the atom types at the interface has a smaller effect on the lattice constants, while it has a greater effect on the interlayer distance because of the different sizes of the atoms at the interface. The stability of the HSs is analysed by considering the layer binding energy *E*_b_ and the results of AIMD simulations at 300 K. The layer binding energy *E*_b_ is defined as the difference between the energy of the heterostructure *E*_HS_ and the sum of the energy of the constituent monolayers *E*_ML1,2_: *E*_b_ = *E*_HS_ − (*E*_ML1_ + *E*_ML2_). The calculated *E*_b_ for 1T-PtSSe/2H-WSSe and 1T-PtSSe/2H-WSeTe HS are tabulated in Table 2, while for the 1T-PtSSe/2H-WSTe systems the *E*_b_ values are given in Table S5 of the SI. The constituent monolayers (PtSSe and WXY) already show synthesis feasibility by cohesive energy calculations,^45,50^ and were found to be dynamically stable by phonon dispersion calculations^45,49,50,70^ The calculated layer binding energy values for 1T-PtSSe/2H-WSTe and 1T-PtSSe/2H-WSeTe HSs are negative, which supports the energetic feasibility of bilayer formation. In the case of 1T-PtSSe/2H-WSSe HSs, the values of *E*_b_ are positive, suggesting that layer binding is less favourable and can be obtained under suitable synthesis conditions; for this reason, we do not further consider the 1T-PtSSe/2H-WSSe HSs in what follows.

**Table 1:** Optimized lattice constants (*a* = *b*) and interlayer distance *d* [Å] for 1T-PtSSe/2H-WSSe (PtWSSe), 1T-PtSSe/2H-WSTe (PtWSTe) and 1T-PtSSe/2H-WSeTe (PtWSeTe) HSs in different interface configurations for stacking order S1

Atom facing types	PtWSSe		PtWSTe		PtWSeTe	
	Lattice constant	*d*	Lattice constant	*d*	Lattice constant	*d*
IC1	3.4063	3.62 (Se–S)	3.4773	3.60 (Se–S)	3.5169	3.69 (Se–Se)
IC2	3.4061	3.72 (Se–Se)	3.4763	3.91 (Se–Te)	3.5160	3.90 (Se–Te)
IC3	3.4069	3.65 (S–Se)	3.4773	3.80 (S–Te)	3.5167	3.79 (S–Te)
IC4	3.4071	3.57 (S–S)	3.4779	3.55 (S–S)	3.5175	3.62 (S–Se)

**Table 2:** Layer binding energy *E*_b_ [eV] for 1T-PtSSe/2H-WSSe (PtWSSe) and 1T-PtSSe/2H-WSeTe (PtWSeTe) HSs with different ICs and stacking orders

Stacking order	PtWSSe				PtWSeTe			
	IC1 (Se–S)	IC2 (Se–Se)	IC3 (S–Se)	IC4 (S–S)	IC1 (Se–Se)	IC2 (Se–Te)	IC3 (S–Te)	IC4 (S–Se)
S1	0.338	0.336	0.342	0.353	−0.059	−0.058	−0.058	−0.049
S2	0.261	0.259	0.246	0.263	−0.162	−0.152	−0.182	−0.167
S3	0.252	0.254	0.255	0.265	−0.161	−0.150	−0.161	−0.154
S4	0.260	0.255	0.256	0.274	−0.153	−0.157	−0.168	−0.144
S5	0.268	0.268	0.262	0.268	−0.150	−0.128	−0.153	−0.160

**Fig. 3:**
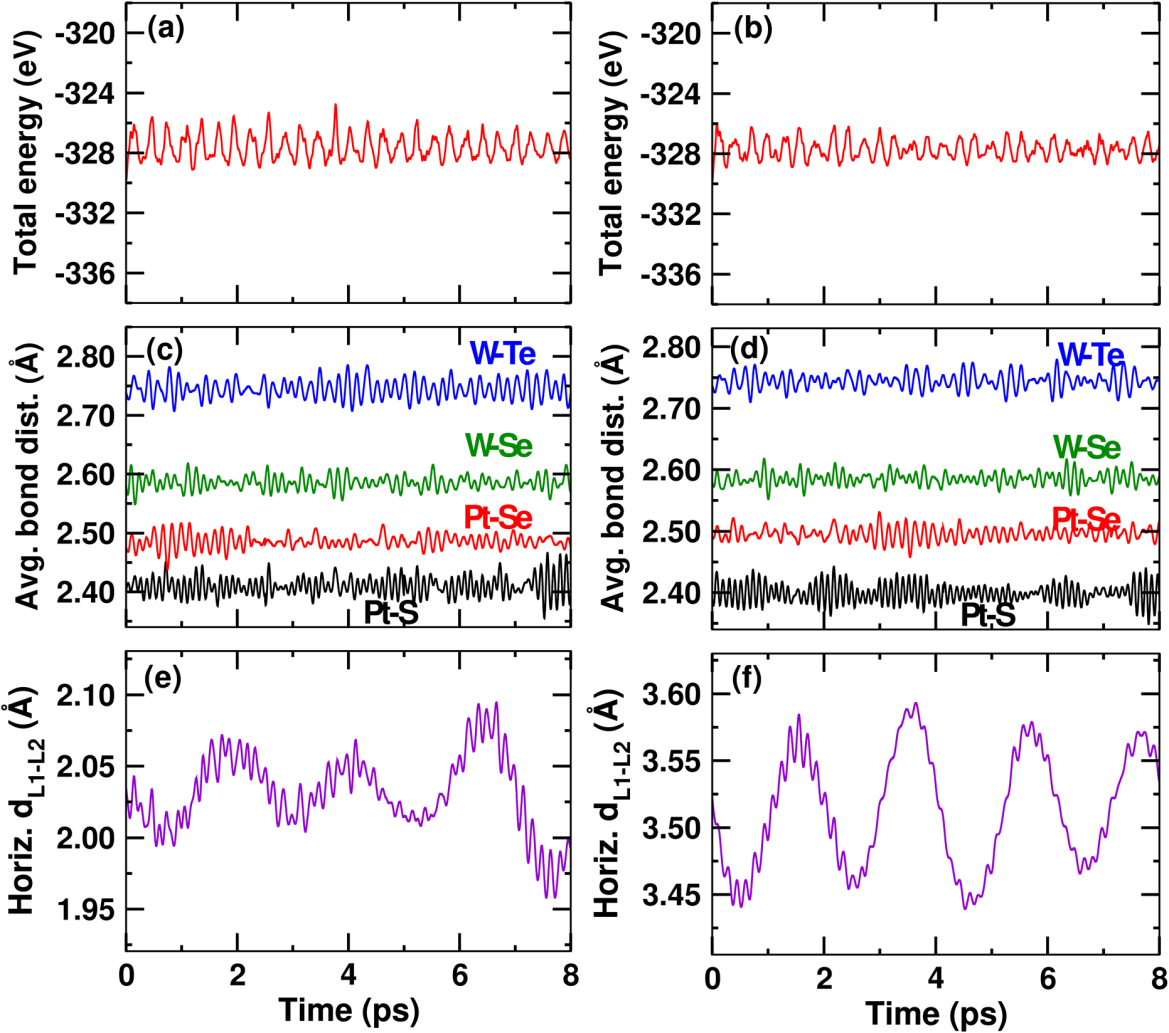
Variation of total energy of the system, average bond distance (avg. bond dist.) and horizontal distance between layers (horizon. *d*_L1–L2_) as a function of time. Left (a, c and e) and right (b, d and f) panels show the results for 1T-PtSSe/2H-WSeTe-IC1-S2 and 1T-PtSSe/2H-WSeTe-IC4-S2 systems, respectively.

Results of AIMD simulations at 300 K are shown in Fig. 3. The variation of the total energy of 1T-PtSSe/2H-WSeTe-IC1-S2 and 1T-PtSSe/2H-WSeTe-IC4-S2 HSs as function of time is shown in Fig. 3a and b, respectively. To check for possible breaking of the atomic bonds, we monitored the average bond distance between the metal and first neighbouring chalcogenide atoms over the simulation time (Fig. 3c and d). For both HSs, less than 0.1 Å variation in the bond length is observed. Further, we calculate standard deviation of these bond distances and the average over simulation time which are reported in Table S6 of SI. A standard deviation of less than 0.02 Å is observed for any configurations considered. In addition, the possibility of relative layer sliding compared to the initial stacking order is checked (Fig. 3e and f). To this aim, we monitor the horizontal distance between the metal atoms of upper (WXY) and lower (PtSSe) layers. The horizontal distance at a given time step is calculated from the average of *a* and *b* coordinates of upper and lower layer metal atoms. The plots show a change of less than 0.1 Å in the horizontal distance from the initial position, which indicates that the initial stacking order is stable; similar plots for 1T-PtSSe/2H-WSeTe-IC2-S2 HS are shown in Fig. S4 of the SI. These results suggest that the HSs are stable at operating temperature.

The *ab initio* calculations of layer binding energy and AIMD simulations in the present work suggest stability of PtSSe–WSeTe based heterostructures. The monolayers of the TMDCs PtS_2_,^71^ PtSe_2_,^72^ WS_2_ (ref. 73) and WSe_2_ (ref. 74) have been reported to be synthesised experimentally. In literature, experimental synthesis of Janus MoSSe has been reported by thermal selenisation of its non-Janus counterpart MoS_2_.^43,75^ Further, heterostructures based on them can be realised by techniques such as mechanical stacking.^76^ Therefore, applying similar experimental strategies suggests that the synthesis of a Janus PtSSe or WSeTe monolayer is highly feasible.

### 3.2 Electronic properties

**Fig. 4:**
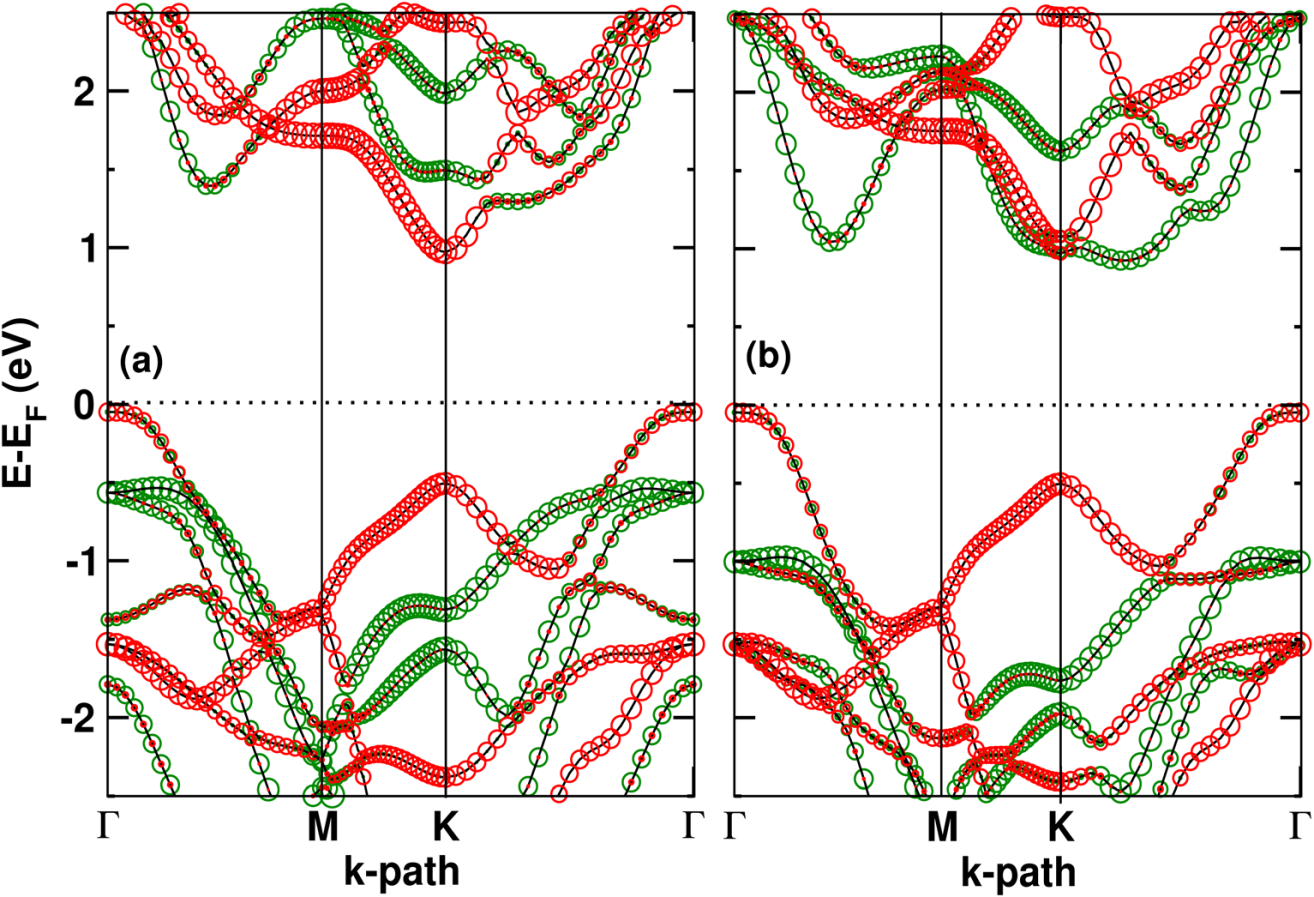
Electronic band structures with their layer projected band characters of (a) 1T-PtSSe/2H-WSeTe-IC1-S2 (b) 1T-PtSSe/2H-WSeTe-IC4-S2 systems. Red and green circles represent projection onto WSeTe and PtSSe component monolayers, respectively; larger circles indicate larger component contributions.

In this section, we discuss the electronic structure of selected candidate HS configurations (ICs and stacking orders) which can show photocatalytic activity. We individuate the type of band gap, value of band gap and the band edge positions in these HSs, which are useful features for photocatalytic redox reactions. Fig. 4 shows the electronic structure of 1T-PtSSe/2H-WSeTe-IC1-S2 and 1T-PtSSe/2H-WSeTe-IC4-S2 HSs along with their layer projected band structure obtained using HSE06 functional. To check the location of band edges (CBM and VBM) and verify the type of band alignment, we consider the atom-projected band structures onto component monolayer character. The layer character of an electronic band is obtained as the sum of the atom-projected character of each atom forming the layer. The 1T-PtSSe/2H-WSeTe HS has Se–Se atom facing in IC1 and S–Se atom facing in IC4 configurations at the interface; both HSs are indirect band gap semiconductors. The values of the band gap *E*_g_ for 1T-PtSSe/2H-WSeTe in IC1-S2 and IC4-S2 configurations are 1.02 eV and 0.97 eV, respectively (Table 3). For both the HSs, the valence band maximum (VBM) is located at the *Γ*-point. The conduction band minimum (CBM) is realised at the *K*-point for IC1-S2 while it is slightly away from the *K*-point along *Γ*–*K* direction (closer to the *K*-point) for IC4-S2 configuration. In the case of 1T-PtSSe/2H-WSeTe-IC4-S2, two other minima are observed: one is located at the *K*-point, close to the CBM with an energy difference of less than ∼10 meV and another along the *Γ*–*M* path realising a band gap of 1.05 eV. These minima are also expected to contribute to the carrier (electron–hole pair) generation during photon absorption. With the availability of multiple minima with close energy values, high carrier generation is expected in this HS. The direct gap widths in case of 1T-PtSSe/2H-WSeTe for IC1-S2 and IC4-S2 configurations are ∼1.47 and ∼1.43 eV, respectively.

**Table 3:** Band gap, band alignment type, vacuum level, valence band edge *E*_VBM_ and conduction band edge *E*_CBM_ with respect to vacuum level for 1T-PtSSe/2H-WSeTe (PtWSeTe)-IC1-S2 and 1T-PtSSe/2H-WSeTe (PtWSeTe)-IC4-S2 candidates. All the energies are in eV

	*E*_g_	Band alignment	Vacuum level (PtSSe side)	Vacuum level (WSeTe side)	*E*_VBM_	*E*_CBM_
PtWSeTe-IC1-S2	1.02	Type-I	5.27	3.63	−4.35	−3.33
PtWSeTe-IC4-S2	0.97	Type-II	4.60	4.27	−4.54	−3.89

From Fig. 4a, we can see that VBM (*Γ*-point) and CBM (*K*-point) own WSeTe character, suggesting straddled type of alignment; thus, the 1T-PtSSe/2H-WSeTe system with atom facing type IC2 and stacking S2 has type-I band alignment. For 1T-PtSSe/2H-WSeTe (Fig. 4b), it can be observed that the VBM has WSeTe character while CBM has PtSSe character, suggesting this HS in IC4-S2 configuration is of type-II (staggered) band alignment. The presence of VBM and CBM in two separate layers is beneficial to generate carriers in two separate layers and is then helpful in reducing carrier recombination. Also, here we note that changing the atom facing type is a useful way to tune the band alignment type between type-I and type-II.

Lastly, we here provide a brief discussion on the electronic structure of 1T-PtSSe/2H-WSTe HSs. On changing the atom facing type and stacking orders, the 1T-PtSSe/2H-WSTe HSs show electronic band structures ranging from metallic to semiconducting with the highest band gap value equal to 0.84 eV. The electronic band structure for a case of 1T-PtSSe/2H-WSTe in IC1 atom facing type for the most stable stacking order S3 is shown in Fig. S6 of SI. The VBM and CBM are realised at the *Γ*- and *K*-point, respectively, while the band gap is found to be indirect with a width equal to 0.57 eV. We do not further explore and discuss these HSs for photocatalytic applications as their band gap are lower for efficient absorption of visible light; however, these HSs can be explored for other applications such as thermoelectric energy conversion and infrared photovoltaics where narrow band gap semiconductors are useful.^77–82^

### 3.3 Photocatalytic activity

**Fig. 5:**
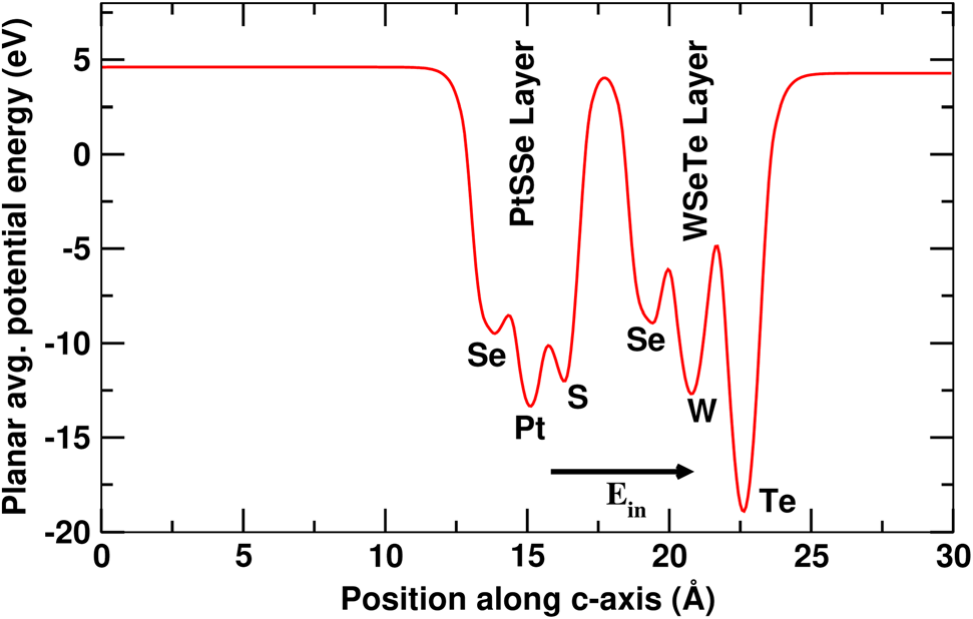
Planar averaged electrostatic potential along the *c*-axis for 1T-PtSSe/2H-WSeTe (PtWSeTe)-IC4-S2. The direction of the internal electric field *E*_in_ is shown by the arrow in the figure.

In this section, we discuss the band edge alignments in the selected HSs for HER, OER and CO_2_ reduction applications. The difference in electronegativity of the chalcogen atoms in the considered Janus HSs generates a dipole moment and an internal electric field. Moreover, because of the electronegativity difference, the two sides (upper WXY and lower PtSSe sides—Fig. 1) of the HS bilayer have different vacuum levels. In the cases where the VBM and CBM are located in two different layers, we align the VBM and CBM edges with respect to the vacuum levels of the respective sides following the method by X. Li *et al.*^83^ We present the *ab*-planar averaged electrostatic potential along *c*-direction for 1T-PtSSe/2H-WSeTe-IC4-S2 as a case, which possesses a type-II band gap, in order to show the vacuum potential differences arising due to the chalcogen atoms of different electronegativity in the considered HSs. From Fig. 5, difference in vacuum potentials at the PtSSe side (4.60 eV) and at the WSeTe side (4.27 eV) and the asymmetric nature of the potential peaks can be observed. This asymmetric nature of the potential curves indicates an internal electric field arising due to the presence of chalcogen atoms of different electronegativity. The direction of the internal field *E*_in_ is from PtSSe (lower) layer to WSeTe (upper) layer as shown in Fig. 5, with an estimated magnitude of ∼8 V Å^−1^.

The hydrogen and oxygen evolution reactions occur thanks to the following reduction and oxidization processes:

1 2H^+^ + 2e^−^ → H_2_

2
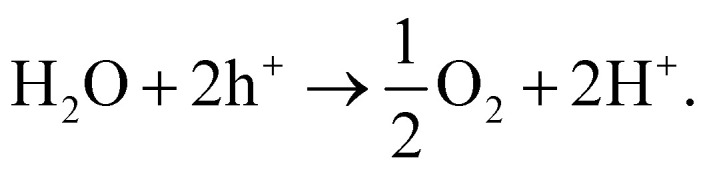


The reduction and oxidation potential of the above reactions at pH = 0 are −4.44 eV and −5.67 eV, respectively, with respect to the vacuum level.^7^ Similarly, the photoreduction of CO_2_ to HCOOH, CO and other molecules takes place at pH = 7 through the following multielectron steps:^84,85^

3 CO_2_ + 2H^+^ + 2e^−^ → HCOOH

4 CO_2_ + 2H^+^ + 2e^−^ → CO + H_2_O

5 CO_2_ + 4H^+^ + 4e^−^ → HCHO + H_2_O

6 CO_2_ + 6H^+^ + 6e^−^ → CH_3_OH + H_2_O

7 CO_2_ + 8H^+^ + 8e^−^ → CH_4_ + 2H_2_O.

The reduction potentials for reactions in eqn (3)–(7) with respect to vacuum level are −3.89, −3.97, −4.02, −4.12 and −4.26 eV, respectively, which are obtained using the relation in ref. 86. For reduction reaction to take place, the CBM edge should be higher in energy than the reduction potential; similarly for oxidation to take place the VBM edge should be lower in energy than the oxidation potential. To assess the photocatalytic redox activities of the selected HSs, we then focus on the CBM and VBM edge positions aligned with respect to the vacuum level. Table 3 shows candidates which can act as photocatalysts for HER and CO_2_ reduction reactions. In the table, the valence (*E*_VBM_) and conduction (*E*_CBM_) band edge energies are obtained by aligning them with respect to the vacuum level. In case of candidates with band edges located in the same layer, the band edges are aligned with respect to the vacuum level of the component monolayer side in which both edges (VBM and CBM) are located. By inspecting the table, we can see that both the 1T-PtSSe/2H-WSeTe-IC1-S2 and 1T-PtSSe/2H-WSeTe-IC4-S2 HS can act as photocatalysis for HER with *E*_CBM_ values higher than water reduction potential of −4.44 eV. The type-II nature of 1T-PtSSe/2H-WSeTe-IC4-S2 can be an advantage for efficient carrier generation in separate layers for HER with multiple minima contributing to higher number of conduction band electrons. Instead, the efficiency of 1T-PtSSe/2H-WSeTe-IC1-S2 can be lower because of the type-I nature of the alignment, favouring possible carrier recombination of electrons–hole pairs generated in the same layer. We find that these HSs do not show OER with *E*_VBM_ edges higher in energy than the water oxidation potential of −5.67 eV. The PtSSe monolayer has a higher band gap of 2.19 eV and shows both HER and OER properties compared to these HSs.^49,50^ Another component, the WSeTe monolayer, with a band gap of 1.79 eV, shows only OER properties.^45^ A summary of photocatalytic activity of different types of Janus 2D materials can be found in ref. 87. Concerning the CO_2_ reduction capabilities, by comparing the potentials of multielectron reduction of CO_2_ in aqueous medium (eqn (3)–(7)) and the CBM energies from Table 3, we find that 1T-PtSSe/2H-WSeTe-IC1-S2 can be a suitable candidate. With its conduction band edge above these reduction potential, it can act as photocatalysts for CO, HCHO, CH_3_OH and CH_4_ conversion of CO_2_. The other candidate 1T-PtSSe/2H-WSeTe-IC4-S2 has also the suitable conduction band edge to drive the reduction reactions of CO_2_ except for the HCOOH conversion. However, for some of the above reactions, the overpotentials (the energy difference between the reduction (oxidation) potential and CBM (VBM)) to drive the reactions can be low, requiring the need to supply additional energy by an external electric potential difference. Such materials can then be suitable candidates for photoelectrocatalysts in HER and CO_2_ reduction reactions.

**Fig. 6:**
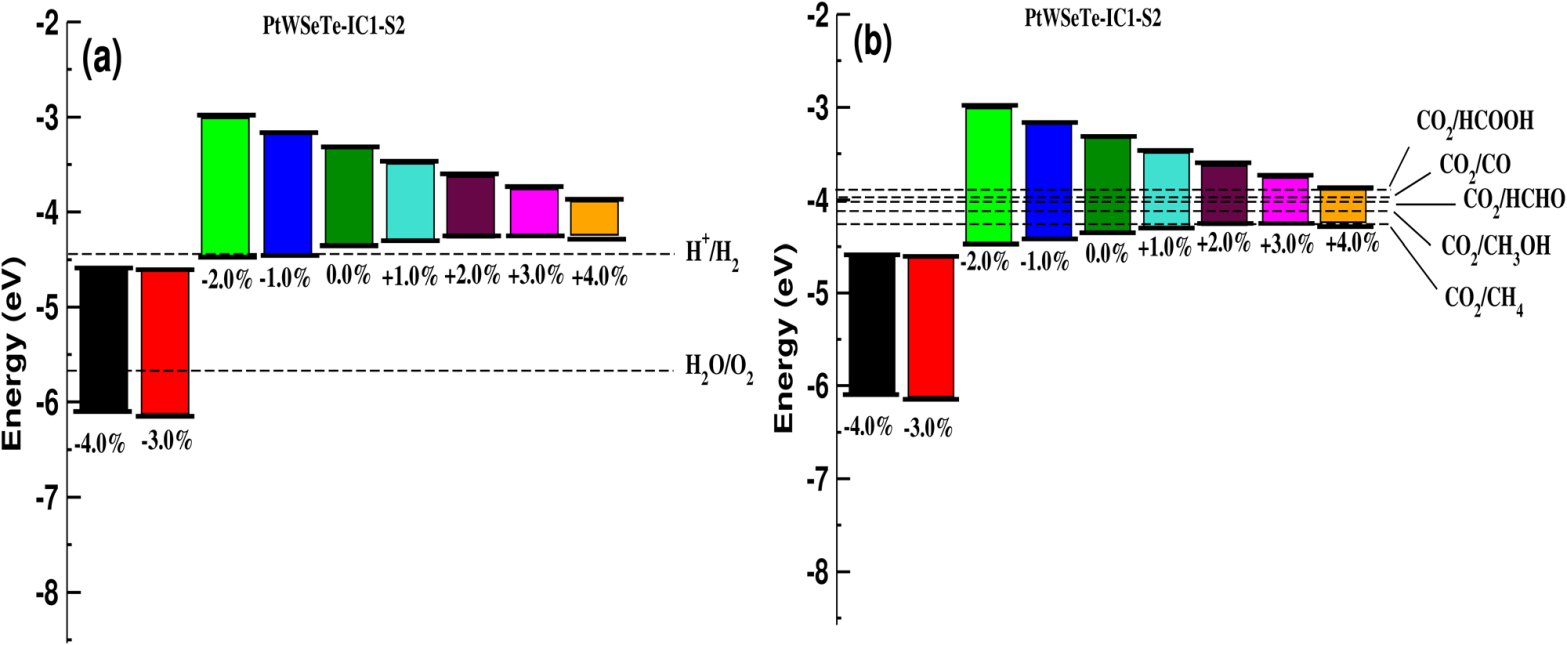
Alignment of band edge positions of strained 1T-PtSSe/2H-WSeTe (PtWSeTe)-IC1-S2 heterostructures with (a) water redox potential and (b) CO_2_ reduction potentials.

To get a better picture of the band edge alignments we provide energy level diagrams in which we report the alignment of the band edge positions and redox potentials with respect to the vacuum level (Fig. 6). HER and OER activities can be estimated from Fig. 6a, while CO_2_ reduction activities from Fig. 6b. It can be observed that the CBM position of 1T-PtSSe/2H-WSeTe IC1-S2 and 1T-PtSSe/2H-WSeTe IC4-S2 favours the donation of electrons to H^+^ ions for the reduction reaction. Fig. 6b suggests that 1T-PtSSe/2H-WSeTe IC1-S2 and 1T-PtSSe/2H-WSeTe IC4-S2 HSs have conduction band edge positions favourable to drive the CO_2_ reduction reaction to useful molecules. Moderate values of overpotentials (∼0.5–1.0 eV) can be observed for the two candidates to drive these reactions, except for the case of CO_2_ reduction with 1T-PtSSe/2H-WSeTe-IC4-S2.

**Fig. 7:**
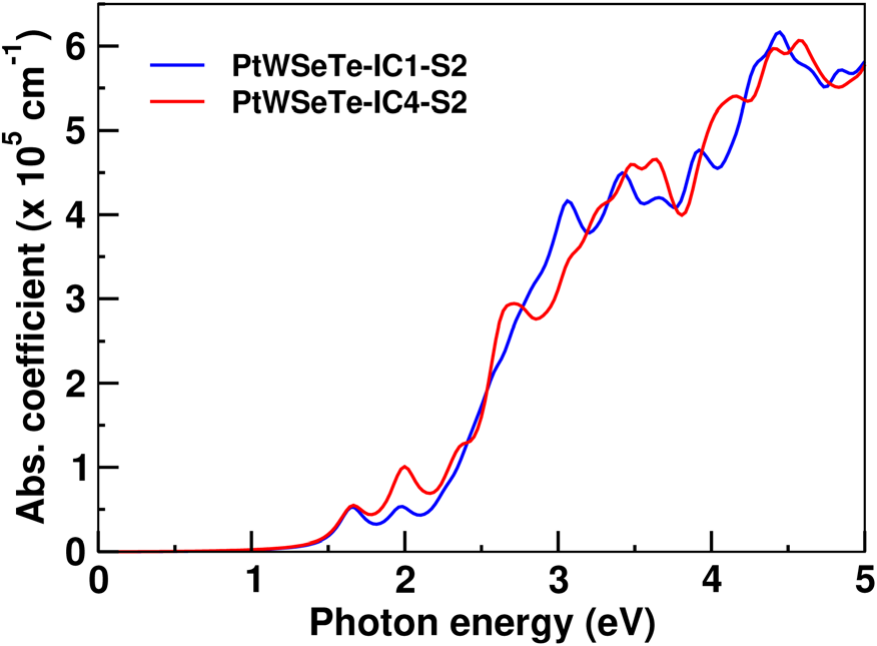
Calculated optical absorption coefficient *α* for 1T-PtSSe/2H-WSeTe (PtWSeTe)-IC1-S2 and 1T-PtSSe/2H-WSeTe (PtWSeTe)-IC4-S2 heterostructures.

In addition to possessing a favourable band edge alignment, a photocatalytic material needs to show a good incident light absorption property for overall efficiency. Therefore, we calculate the optical absorption coefficient *α* as a function of incident photon energy for the HSs which show favourable band edge alignments for redox reactions, namely the 1T-PtSSe/2H-WSeTe-IC1-S2 and 1T-PtSSe/2H-WSeTe-IC4-S2 systems (Fig. 7). *α* is obtained by using the real and imaginary parts of the complex dielectric function from the HSE06 calculations. The spectra show absorption peaks in the visible region because of direct transitions for both candidates. The 1T-PtSSe/2H-WSeTe-IC1-S2 system shows higher absorption of incident photons around 2 eV while, 1T-PtSSe/2H-WSeTe-IC4-S2 shows higher values of the absorption coefficient for incident photon energy around 3 eV. The predicted absorption spectra suggest these HSs can utilise visible part of the solar spectrum by direct transitions then drive photocatalytic redox reactions in an efficient way. Exciton binding that arises as a result of the interaction between photo-excited electron–hole pairs is an important quantity that affects the optical properties of 2D materials,^88–94^ and should be treated at the GW^95^ or BSE^96^ level of theory. Since the corresponding computational load is high, this effect needs to be part of a more dedicated future study; however, we believe that the HSE06 functional here used can already provide useful insights for the scope of the present study.

### 3.4 Band edge tuning by strain

Application of strains is one of the techniques used to engineer the positions of band edges of materials.^3,97^ This technique is also helpful in effectively tuning electronic and optical properties of 2D materials.^98–100^ Here, we further study the effect of biaxial strain on the band edges of the HSs and check if they can be made suitable for photocatalytic applications by tuning the band edge positions with respect to redox potentials. We apply biaxial compressive strain in the range [−4, −1]% and tensile strain in the range [+1, +4]% of the initial lattice constants, and calculate the related electronic band structure and vacuum potentials using the HSE06 functional. Here, we present and discuss only those HS configurations that showed suitable band edge alignment for photocatalytic activity, namely 1T-PtSSe/2H-WSeTe-IC1-S2 and 1T-PtSSe/2H-WSeTe-IC2-S2.

Understanding the stability under stress is important to assess the stiffness of the photocatalyst under strain, as strain engineering is one of the ways to tune the electronic and optical properties of a material. Therefore, we evaluate the dynamic stability by subjecting the lattice to a biaxial strain from −4.0% to +4.0% in step of 1.0% and calculating the second order elastic constants *C*_*ij*_ using the energy-strain relation as implemented in the VASPKIT package.^69^ We calculated the *C*_*ij*_ coefficients for the two strained systems which show photcatalytic activity namely, 1T-PtSSe/2H-WSeTe-IC1-S2 and 1T-PtSSe/2H-WSeTe-IC2-S2. For the 2D hexagonal systems the independent *C*_*ij*_ coefficients are *C*_11_ and *C*_12_. The restriction on stability criteria for *C*_*ij*_ in case of 2D hexagonal system are (i) *C*_11_ > 0 and (ii) *C*_11_ > |*C*_12_|.^101^ The calculated two independent elastic constants for the 1T-PtSSe/2H-WSeTe-IC1-S2 system are *C*_11_ = 164.02 and *C*_12_ = 41.63 N m^−1^. For 1T-PtSSe/2H-WSeTe-IC2-S2, the values of *C*_11_ and *C*_12_ are 162.77 and 40.69 N m^−1^, respectively. These *C*_*ij*_ elements satisfy the stability criteria, then supporting the conclusion that the two systems are stable under applied strain.

The general variation of band gap for the full range of applied strains for these HSs is reported in Table 4. The application of tensile strain reduces the band gap width in both systems, while compressive strain increases the gap width for 1T-PtSSe/2H-WSeTe-IC1-S2, for strain up to −3.0% and upto −1.0% for 1T-PtSSe/2H-WSeTe-IC2-S2. The values of band gaps, type of band alignment, vacuum level, band edge positions with respect to vacuum, particularly for those strain percentages which adjust the band edge positions for redox reactions are reported in Table 5. Before discussing the photocatalytic activity of these strained HSs, we give a brief discussion of electronic structure and layer projected band characters of them. The electronic band structure of the pristine (unstrained) 1T-PtSSe/2H-WSeTe-IC2-S2 is shown in Fig. S7 of SI and that of 1T-PtSSe/2H-WSeTe-IC1-S2 is shown in Fig. S5a. The band structures and projected band structure (type of band alignment) plots for the strained HSs are discussed in section “band edge tuning by strain” of SI. Fig. S8 of SI shows the band structures for strained systems which are selected candidates for photocatalytic activity. The layer projected band structures for all the strained systems are shown in Fig. S9 and S10 of SI. We find that strained and pristine 1T-PtSSe/2H-WSeTe-IC1-S2 has mainly type-I band alignment. In the case of PtSeTe-IC2-S2, the pristine system and those with applied compressive strains have type-II band alignment, while the systems under tensile strain show type-I band alignment. The variation of planar averaged electrostatic potential along *c*-axis for the considered range of applied biaxial strain is shown in Fig. S11 of SI. The vacuum potentials at the upper (WSeTe) and lower (PtSeTe) sides of the HSs are found to decrease by the application of biaxial strain from −4.0% to +4.0%. This trend is due to the increase in the lattice parameter and reduced electrostatic interaction among the atoms.

**Table 4:** Band gap [eV] of 1T-PtSSe/2H-WSeTe (PtWSeTe)-IC1-S2 and 1T-PtSSe/2H-WSeTe (PtWSeTe)-IC2-S2 systems at the considered compressive and tensile strain values

Strain (%)	PtWSeTe-IC1-S2	PtWSeTe-IC2-S2
−4.0	1.45	0.99
−3.0	1.50	1.07
−2.0	1.46	1.14
−1.0	1.24	1.22
0.0	1.02	1.21
1.0	0.82	1.18
2.0	0.65	1.00
3.0	0.48	0.82
4.0	0.35	0.67

**Table 5:** Strain, band gap, band alignment, vacuum level, valence band edge energy *E*_VBM_ and conduction band edge energy *E*_CBM_ with respect to vacuum level for 1T-PtSSe/2H-WSeTe (PtWSeTe)-IC1-S2 and 1T-PtSSe/2H-WSeTe (PtWSeTe)-IC2-S2. All the energies are in eV

	Strain (%)	*E*_g_	Band alignment	Vacuum level (PtSSe side)	Vacuum level (WXY side)	*E*_VBM_	*E*_CBM_
PtWSeTe-IC1-S2	−1.0	1.24	Type-I	5.34	3.71	−4.41	−3.17
	−2.0	1.46	Type-I	5.42	3.80	−4.47	−3.01
	−3.0	1.50	Type-I	5.50	3.89	−6.12	−4.61
	−4.0	1.45	Type-I	5.58	3.99	−6.03	−4.58
PtWSeTe-IC2-S2	−1.0	1.22	Type-II	4.92	4.76	−5.72	−4.66
	−2.0	1.14	Type-II	4.99	4.86	−5.76	−4.74

The energy level diagram for all the strained 1T-PtSSe/2H-WSeTe-IC1-S2 and 1T-PtSSe/2H-WSeTe-IC2-S2 systems are shown in Fig. 6 and S12 of SI, respectively. The selected best candidates from the band edge position and redox level alignment diagram for photocatalysis are tabulated in Table 5. From Fig. 6a, comparison of hydrogen reduction potential and the conduction band edges suggests that 1T-PtSSe/2H-WSeTe-IC1-S2 at −1.0 and −4.0% strains can be a potential candidate for HER thanks to a higher *E*_CBM_ compared to the potential for the reaction; however, the efficiency of the reaction might be limited due to the type-I nature of the band alignment. For OER, the comparison of *E*_VBM_ with the oxidation potential of water (−5.67 eV) (Fig. 6a) suggests that strained 1T-PtSSe/2H-WSeTe-IC1-S2 HSs (−3.0 and −4.0% strain) can be candidates for OER; however with limited efficiency due to the type-I nature of the band alignment. The CO_2_ reduction reaction is favourable by the conduction band edge position of strained 1T-PtSSe/2H-WSeTe-IC1-S2 HS (−1.0 and −2.0% strain, Fig. 6b), although the efficiency might be low due to the type-I nature of the band alignment in this material. We note that 1T-PtSSe/2H-WSeTe-IC1-S2 HS at tensile strain values +1.0 to +4.0% show conduction band edges alignment above hydrogen reduction (Fig. 6a) and CO_2_ reduction potential (Fig. 6b). However, these systems are not selected for photocatalysis because of type-I nature of band alignments along with lower band gap (∼0.3–0.8 eV) values for visible light absorption.

The relative alignment of band edge positions of 1T-PtSSe/2H-WSeTe-IC2-S2 strained HSs and redox potentials are graphically represented in Fig. S12 of SI. We find that for OER, the comparison of *E*_VBM_ with the oxidation potential of water (−5.67 eV) suggests that strained 1T-PtSSe/2H-WSeTe-IC2-S2 HSs (−1.0 and −2.0% strain) can be potential candidates with better efficiency thanks to the type-II nature of the band alignment. We observe that valence band edges of strained 1T-PtSSe/2H-WSeTe-IC2-S2 can accept electrons from water molecules, acting as promising candidate for OER. For HER and CO_2_ reduction reactions, it is observed that strained 1T-PtSSe/2H-WSeTe-IC2-S2 HSs do not show favourable band edge alignment for reduction. Finally, we find that the conduction band edge of strained 1T-PtSSe/2H-WSeTe-IC1-S2 has favourable position to donate electrons for HER and CO_2_ reduction reaction. The present study is focused on the crystalline (*i.e.* non-defective) HSs made up of 1T-PtSSe/2H-WSSe and WSeTe as candidates for photocatalytic redox reactions. However, in TMDC based HSs generally anion vacancies are known to occur at the interfaces or interior of the material.^102^ Surface anionic vacancies in photocatalysts usually act as shallow donors while cation vacancies as shallow acceptors.^103^ The presence of vacancies can improve or degrade the performance of photocatalysts depending on the type of vacancies.^104,105^ Defects at the surface can also affect the charge migration on the surface and kinetics of surface reaction.^106^ The built-in electric field is known to enhance carrier separation and increases the availability of surface carriers, thereby improving surface kinetics.^107^ Therefore, exploration of the effect of defects and surface kinetics on the photocatalytic properties of these HSs deserves a dedicated study.

## 4 Summary

In this work, we study three bilayer heterostructures formed by one Janus PtSSe and one WXY monolayer, namely 1T-PtSSe/2H-WSSe, 1T-PtSSe/2H-WSTe and 1T-PtSSe/2H-WSeTe, in the context of photocatalytic applications. Four atom facing types and five different stacking orders are considered. The suitability of the HS configurations for photocatalytic redox reactions is evaluated by aligning the *ab initio* calculated band edges with respect to redox potentials of HER, OER and CO_2_ reduction reactions. We find that 1T-PtSSe/2H-WSeTe-IC1-S2 and 1T-PtSSe/2H-WSeTe-IC4-S2 HSs can be promising candidates for HER and CO_2_ reduction reactions, whereas the type-II band alignment of 1T-PtSSe/2H-WSeTe-IC4-S2 suggests higher reaction efficiency in this material. Optical absorption spectra indicate that both systems exhibit good visible-light absorption. The stability of these candidate HSs is supported by formation energy values, elastic stability criteria and AIMD simulations at 300 K. In addition, biaxial strain engineering is used to tune the band edge positions, in order to favour redox reactions. Under compressive strains of −1.0% and −2.0%, 1T-PtSSe/2H-WSeTe-IC1-S2 emerges as a potential candidate for HER and CO_2_ reduction and for strains of −3.0% and −4.0% as photocatalyst for OER. The 1T-PtSSe/2H-WSeTe-IC2-S2 can act as a potential photocatalyst for OER under compressive strain of −1.0% and −2.0% with type-II band alignment. These results indicate that HSs composed of Janus PtSSe and WSeTe monolayers are promising materials to be explored for photocatalytic applications. Finally, we suggest that 1T-PtSSe/2H-WSTe HSs with narrow band gaps can be explored for use in thermoelectric generator or infrared photovoltaics applications.

## Author contributions

Shivprasad S. Shastri: conceptualisation, data curation, formal analysis, investigation, methodology, validation, visualisation, writing—original draft. Antonio Cammarata: conceptualisation, formal analysis, funding acquisition, investigation, methodology, validation, visualisation, writing—review and editing. Tomas Polcar: funding acquisition, validation, writing—review and editing.

## Conflicts of interest

There are no conflicts to declare.

## Supplementary Material

RA-OLF-D6RA03413F-s001

## Data Availability

Data for this article, including geometries of the reported structures are available at Zenodo repository at https://doi.org/10.5281/zenodo.18399958. Supplementary information (SI): structural properties, stability, electronic properties, band edge tuning by strain, optimized structure files. See DOI: https://doi.org/10.1039/d6ra03413f.
