# Supplementary material for: Ab initio study on photocatalytic properties of PtSSe–WXY Janus heterostructures
Source: RSC Adv. 2026 Jul 3;16(35):36705–16. doi: 10.1039/d6ra03413f (PMC13331670; doi:10.1039/d6ra03413f)
Supplement: RA-016-D6RA03413F-s001 [file RA-016-D6RA03413F-s001.pdf]

Supplementary Information (SI) for RSC Advances.
This journal is © The Royal Society of Chemistry 2026

Journal Name

ARTICLE TYPE

Cite this: DOI: 00.0000/xxxxxxxxxx

Ab initio study on photocatalytic properties of PtSSe-WXY Janus heterostructures — Electronic Supplementary Information

Shivprasad S. Shastri,*[a] Antonio Cammarata,*[a] and Tomas Polcar[b]

Contents

1 Structural Properties 3

2 Stability 5

3 Electronic properties 5

4 Band edge tuning by strain 6

5 Optimized structure files 13
5.1 1T-PtSSe monolayer 13
5.2 2H-WSSe monolayer 13
5.3 2H-WSTe monolayer 13
5.4 2H-WSeTe monolayer 13
5.5 1T-PtSSe/2H-WSSe-IC1-S1 14
5.6 1T-PtSSe/2H-WSSe-IC1-S2 14
5.7 1T-PtSSe/2H-WSSe-IC1-S3 14
5.8 1T-PtSSe/2H-WSSe-IC1-S4 15
5.9 1T-PtSSe/2H-WSSe-IC1-S5 15
5.10 1T-PtSSe/2H-WSSe-IC2-S1 15
5.11 1T-PtSSe/2H-WSSe-IC2-S2 16
5.12 1T-PtSSe/2H-WSSe-IC2-S3 16
5.13 1T-PtSSe/2H-WSSe-IC2-S4 16
5.14 1T-PtSSe/2H-WSSe-IC2-S5 17
5.15 1T-PtSSe/2H-WSSe-IC3-S1 17
5.16 1T-PtSSe/2H-WSSe-IC3-S2 17
5.17 1T-PtSSe/2H-WSSe-IC3-S3 18
5.18 1T-PtSSe/2H-WSSe-IC3-S4 18
5.19 1T-PtSSe/2H-WSSe-IC3-S5 18
5.20 1T-PtSSe/2H-WSSe-IC4-S1 19
5.21 1T-PtSSe/2H-WSSe-IC4-S2 19
5.22 1T-PtSSe/2H-WSSe-IC4-S3 19
5.23 1T-PtSSe/2H-WSSe-IC4-S4 20
5.24 1T-PtSSe/2H-WSSe-IC4-S5 20
5.25 1T-PtSSe/2H-WSTe-IC1-S1 20
5.26 1T-PtSSe/2H-WSTe-IC1-S2 20
5.27 1T-PtSSe/2H-WSTe-IC1-S3 21
5.28 1T-PtSSe/2H-WSTe-IC1-S4 21
5.29 1T-PtSSe/2H-WSTe-IC1-S5 21
5.30 1T-PtSSe/2H-WSTe-IC2-S1 22
5.31 1T-PtSSe/2H-WSTe-IC2-S2 22
5.32 1T-PtSSe/2H-WSTe-IC2-S3 22
5.33 1T-PtSSe/2H-WSTe-IC2-S4 23
5.34 1T-PtSSe/2H-WSTe-IC2-S5 23
5.35 1T-PtSSe/2H-WSTe-IC3-S1 23
5.36 1T-PtSSe/2H-WSTe-IC3-S2 24
5.37 1T-PtSSe/2H-WSTe-IC3-S3 24
5.38 1T-PtSSe/2H-WSTe-IC3-S4 24
5.39 1T-PtSSe/2H-WSTe-IC3-S5 25
5.40 1T-PtSSe/2H-WSTe-IC4-S1 25
5.41 1T-PtSSe/2H-WSTe-IC4-S2 25
5.42 1T-PtSSe/2H-WSTe-IC4-S3 26
5.43 1T-PtSSe/2H-WSTe-IC4-S4 26
5.44 1T-PtSSe/2H-WSTe-IC4-S5 26
5.45 1T-PtSSe/2H-WSeTe-IC1-S1 27
5.46 1T-PtSSe/2H-WSeTe-IC1-S2 27
5.47 1T-PtSSe/2H-WSeTe-IC1-S3 27
5.48 1T-PtSSe/2H-WSeTe-IC1-S4 28
5.49 1T-PtSSe/2H-WSeTe-IC1-S5 28
5.50 1T-PtSSe/2H-WSeTe-IC2-S1 28
5.51 1T-PtSSe/2H-WSeTe-IC2-S2 28
5.52 1T-PtSSe/2H-WSeTe-IC2-S3 29
5.53 1T-PtSSe/2H-WSeTe-IC2-S4 29
5.54 1T-PtSSe/2H-WSeTe-IC2-S5 29
5.55 1T-PtSSe/2H-WSeTe-IC3-S1 30
5.56 1T-PtSSe/2H-WSeTe-IC3-S2 30
5.57 1T-PtSSe/2H-WSeTe-IC3-S3 30
5.58 1T-PtSSe/2H-WSeTe-IC3-S4 31
5.59 1T-PtSSe/2H-WSeTe-IC3-S5 31

[a] *Department of Control Engineering, Faculty of Electrical Engineering, Czech Technical University in Prague, Karlovo Náměstí 13, Prague, Czech Republic. Fax: +420 224 91 8646; Tel: +420 224 35 5713; E-mail: cammaant@fel.cvut.cz, shastshi@fel.cvut.cz*
[b] *Engineering Materials & nCATS, FEE, University of Southampton, SO17 1BJ, Southampton, United Kingdom.*

5.60 1T-PtSSe/2H-WSeTe-IC4-S1 31
5.61 1T-PtSSe/2H-WSeTe-IC4-S2 32
5.62 1T-PtSSe/2H-WSeTe-IC4-S3 32
5.63 1T-PtSSe/2H-WSeTe-IC4-S4 32
5.64 1T-PtSSe/2H-WSeTe-IC4-S5 33

1 Structural Properties

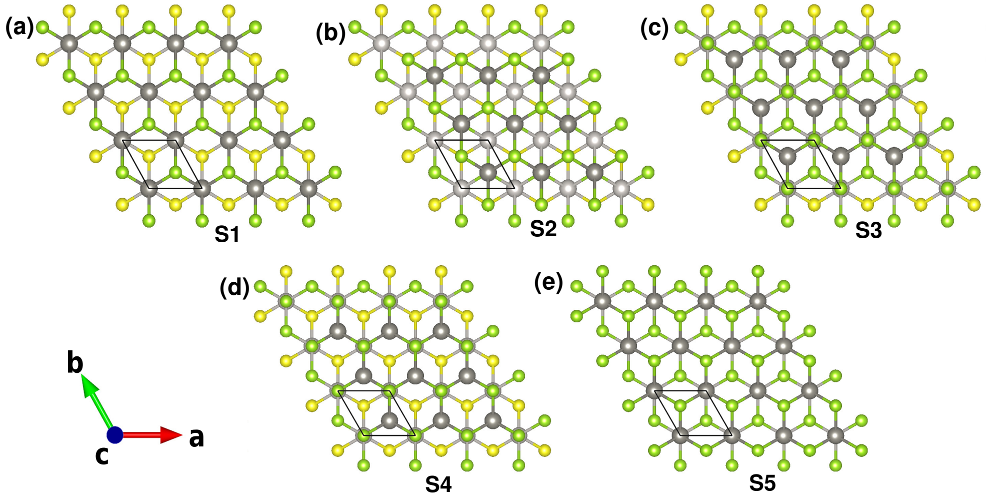

Fig. S1 Top view of the five considered stacking orders S1-S5 shown using 1T-PtSSe/2H-WSSe HS as a representative case in IC1 atom facing type. Black, grey, green and yellow spheres represent W, Pt, S and Se atoms, respectively.

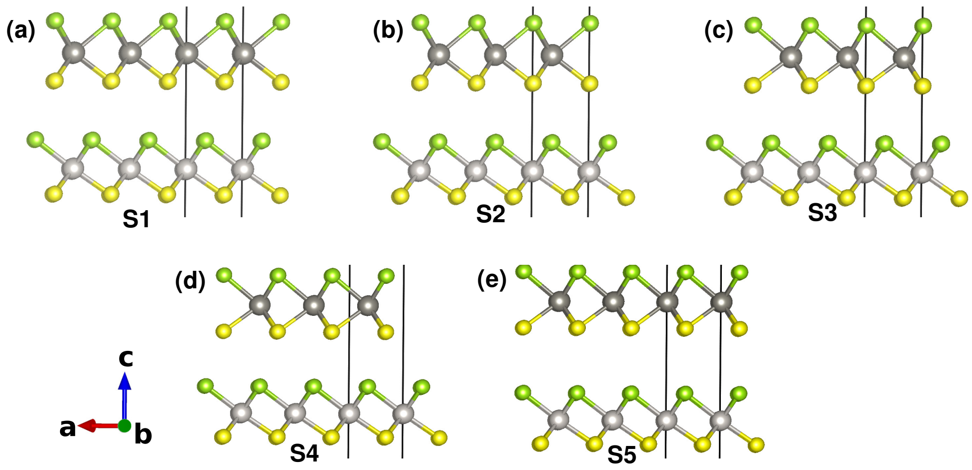

Fig. S2 Side view of the five considered stacking orders S1-S5 shown using 1T-PtSSe/2H-WSSe HS as a representative case in IC1 atom facing type. Black, grey, green and yellow balls represent W, Pt, S and Se atoms, respectively.

Depending on the relative atomic positions of the top and bottom monolayers, different stackings are possible when constructing a bilayer. The top and side views of the considered five stacking orders are shown in Figure S1 and Figure S2, respectively, using 1T-PtSSe/2H-WSSe HS in IC1 atom facing type as a representative case. Similarly, the same five stacking orders are considered in 1T-PtSSe/2H-WSTe and 1T-PtSSe/2H-WSeTe HSs for all four ICs. In both S1 and S5 stacking orders, alignment of WXY layer (upper layer) and PtSSe layer (lower layer) is such that the metal atoms W and Pt are on top of each other. They differ only in the positions of the chalcogen atoms of the WXY layer with respect to the chalcogen atoms of the PtSSe layer. In stacking order S2, the alignment is such that W (chalcogen) atoms of the WXY layer are on top of upper chalcogen atoms (lower chalcogen atoms) of the PtSSe layer. Similarly in S3, W atoms (chalcogen atoms) of the WXY layer are on top of lower chalcogen atoms (Pt atoms) of the PtSSe layer. The stacking order S4 differs from S2 such that the chalcogen atoms of the WXY layer are on top of Pt atoms of the PtSSe layer. The 3D perspective of these arrangements considered is shown in Figure S3.

Optimized lattice constants and interlayer distances d for stacking orders S2-S5 for all the ICs are reported in Tables Table S1-Table S4. Here, d is the distance between the chalcogen atoms at the interface of a HS. The tables show that varying the interfacial atom types has little influence on the lattice constants but has greater effects on the interlayer distance due to differences in atomic size.

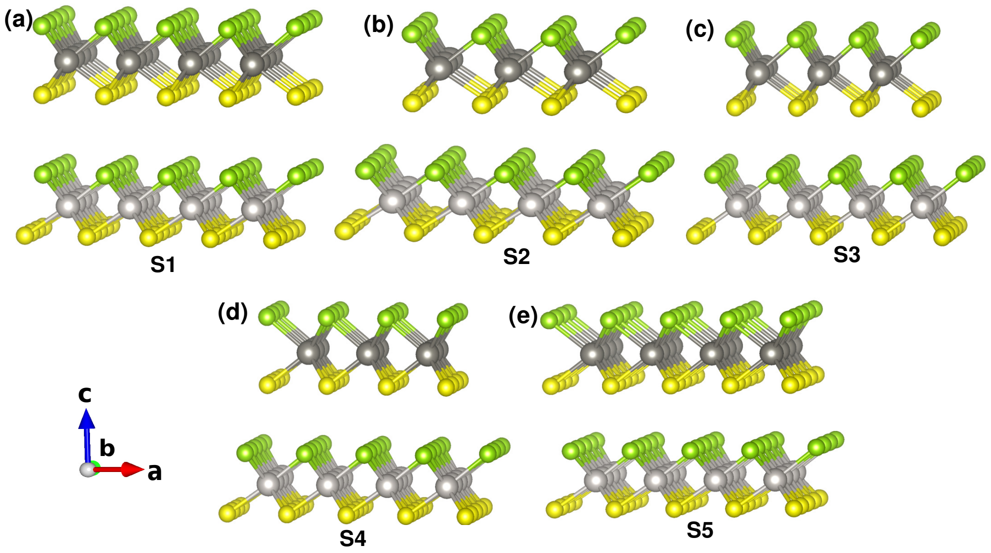

Fig. S3 3D perspective of the five considered stacking orders S1-S5 shown using 1T-PtSSe/2H-WSSe HS as a representative case in IC1 atom facing type. Black, grey, green and yellow spheres represent W, Pt, S and Se atoms, respectively.

Table S1 Optimized lattice constants ($a = b$) and interlayer distance d [Å] of 1T-PtSSe/2H-WSSe (PtWSSe), 1T-PtSSe/2H-WSTe (PtWSTe) and 1T-PtSSe/2H-WSeTe (PtWSeTe) HSs in different interface configurations and stacking order S2.

Atom facing types	PtWSSe		PtWSTe		PtWSeTe	
	Lattice constant	d	Lattice constant	d	Lattice constant	d
IC1	3.4121	3.05 (Se-S)	3.4833	2.98 (Se-S)	3.5244	3.00 (Se-Se)
IC2	3.4123	3.16 (Se-Se)	3.4832	3.30 (Se-Te)	3.5238	3.23 (Se-Te)
IC3	3.4123	2.95 (S-Se)	3.4840	3.00 (S-Te)	3.5241	2.94 (S-Te)
IC4	3.4114	2.91 (S-S)	3.4821	2.86 (S-S)	3.5223	2.84 (S-Se)

Table S2 Optimized lattice constants ($a = b$) and interlayer distance d [Å] of 1T-PtSSe/2H-WSSe (PtWSSe), 1T-PtSSe/2H-WSTe (PtWSTe) and 1T-PtSSe/2H-WSeTe (PtWSeTe) HSs in different interface configurations and stacking order S3.

Atom facing types	PtWSSe		PtWSTe		PtWSeTe	
	Lattice constant	d	Lattice constant	d	Lattice constant	d
IC1	3.4114	3.00 (Se-S)	3.4825	2.93 (Se-S)	3.5228	3.01 (Se-Se)
IC2	3.4113	3.14 (Se-Se)	3.4819	3.32 (Se-Te)	3.5221	3.26 (Se-Te)
IC3	3.4114	3.01 (S-Se)	3.4829	3.15 (S-Te)	3.5221	3.10 (S-Te)
IC4	3.4111	2.90 (S-S)	3.4817	2.85 (S-S)	3.5220	2.90 (S-Se)

Table S3 Optimized lattice constants ($a = b$) and interlayer distance d [Å] of 1T-PtSSe/2H-WSSe (PtWSSe), 1T-PtSSe/2H-WSTe (PtWSTe) and 1T-PtSSe/2H-WSeTe (PtWSeTe) HSs in different interface configurations and stacking order S4.

Atom facing types	PtWSSe		PtWSTe		PtWSeTe	
	Lattice constant	d	Lattice constant	d	Lattice constant	d
IC1	3.4089	3.07 (Se-S)	3.4794	3.04 (Se-S)	3.5194	3.09 (Se-Se)
IC2	3.4092	3.16 (Se-Se)	3.4795	3.28 (Se-Te)	3.5191	3.25 (Se-Te)
IC3	3.4086	3.05 (S-Se)	3.4788	3.11 (S-Te)	3.5177	3.09 (S-Te)
IC4	3.4084	3.00 (S-S)	3.4791	2.98 (S-S)	3.5187	3.00 (S-Se)

Table S4 Optimized lattice constants ($a = b$) and interlayer distance d [Å] of 1T-PtSSe/2H-WSSe (PtWSSe), 1T-PtSSe/2H-WSTe (PtWSTe) and 1T-PtSSe/2H-WSeTe (PtWSeTe) HSs in different interface configurations and stacking order S5.

Atom facing types	PtWSSe		PtWSTe		PtWSeTe	
	Lattice constant	d	Lattice constant	d	Lattice constant	d
IC1	3.4135	3.06 (Se-S)	3.4864	2.93 (Se-S)	3.5268	3.01 (Se-Se)
IC2	3.4125	3.23 (Se-Se)	3.4825	3.44 (Se-Te)	3.5234	3.38 (Se-Te)
IC3	3.4144	3.01 (S-Se)	3.4852	3.18 (S-Te)	3.5264	3.07 (S-Te)
IC4	3.4147	2.86 (S-S)	3.4868	2.73 (S-S)	3.5282	2.76 (S-Se)

2 Stability

Table S5 The layer binding energy E_b in eV for 1T-PtSSe/2H-WSTe (PtWSTe) HSs with different ICs and stacking orders.

Stacking order	PtWSTe			
	IC1 (Se-S)	IC2 (Se-Te)	IC3 (S-Te)	IC4 (S-S)
S1	0.060	0.059	0.053	0.077
S2	-0.031	-0.026	-0.056	-0.023
S3	-0.037	-0.025	-0.040	-0.020
S4	-0.024	-0.034	-0.051	-0.005
S5	-0.028	-0.003	-0.027	-0.024

Table S6 The average bond distance d_{avg} [Å] and standard deviation of bond distance σ_d [Å] for 1T-PtSSe/2H-WSeTe (PtWSeTe) and 1T-PtSSe/2H-WSTe (PtWSTe) HSs in various configurations.

Bond type	PtWSeTe-IC1-S2		PtWSeTe-IC4-S2		PtWSeTe-IC2-S2		PtWSTe-IC1-S3	
	d_{avg}	σ_d	d_{avg}	σ_d	d_{avg}	σ_d	d_{avg}	σ_d
Pt-S	2.4095	0.0161	2.3990	0.0148	2.4081	0.0127	2.3962	0.0103
Pt-Se	2.4857	0.0115	2.4959	0.0106	2.4828	0.0120	2.4853	0.0154
W-Se	2.5855	0.0115	2.5850	0.0102	2.5895	0.0100	–	–
W-Te	2.744	0.0160	2.7441	0.0125	2.7464	0.0123	2.7486	0.0183
W-S	–	–	–	–	–	–	2.4720	0.0117

The layer binding energy E_b for 1T-PtSSe/2H-WSTe in different atom facing types (IC) and stacking orders are presented in Table S5. E_b values are negative for all stacking orders except S1, indicating that the formation of the bilayer is energetically favourable. The total energy variation, average bond distance and horizontal distance between layers as a function of simulation time are shown for 1T-PtSSe/2H-WSTe-IC1-S3 and 1T-PtSSe/2H-WSeTe-IC2-S2 HSs in Figure S4. The variation in the average bond distance between the metal and chalcogen atoms is less than 0.1 Å and the standard deviation is less than 0.2 Å (Table S6). The change in the horizontal distance between the layers (max $\sim$0.5 Å) suggests no significant relative layer sliding with respect the initial stacking order. Thus, results reported in Figure S4 indicate that 1T-PtSSe/2H-WSTe-IC1-S3 and 1T-PtSSe/2H-WSeTe-IC2-S2 systems are thermally stable.

3 Electronic properties

The electronic structure of 1T-PtSSe/2H-WSeTe-IC1-S2, 1T-PtSSe/2H-WSeTe-IC4-S2 HSs and 1T-PtSSe/2H-WSTe HS in IC1-S3 configuration obtained from the HSE06 hybrid functional calculations are shown in Figure S5a, Figure S5b and Figure S6, respectively . It is an indirect band gap semiconductor with gap value of 0.57 eV. The VBM is at the Γ-point and the CBM is realised at the K-point. The electronic band structure of 1T-PtSSe/2H-WSeTe in IC2-S2 configuration without external strain (Figure S7) shows that this HS has indirect band gap with width equal to 1.22 eV. 1T-PtSSe/2H-WSeTe in IC2-S2 configuration has other conduction band minima with close energy values along Γ-K and Γ-M directions, suggesting enhanced efficiency for electron photoexcitation.

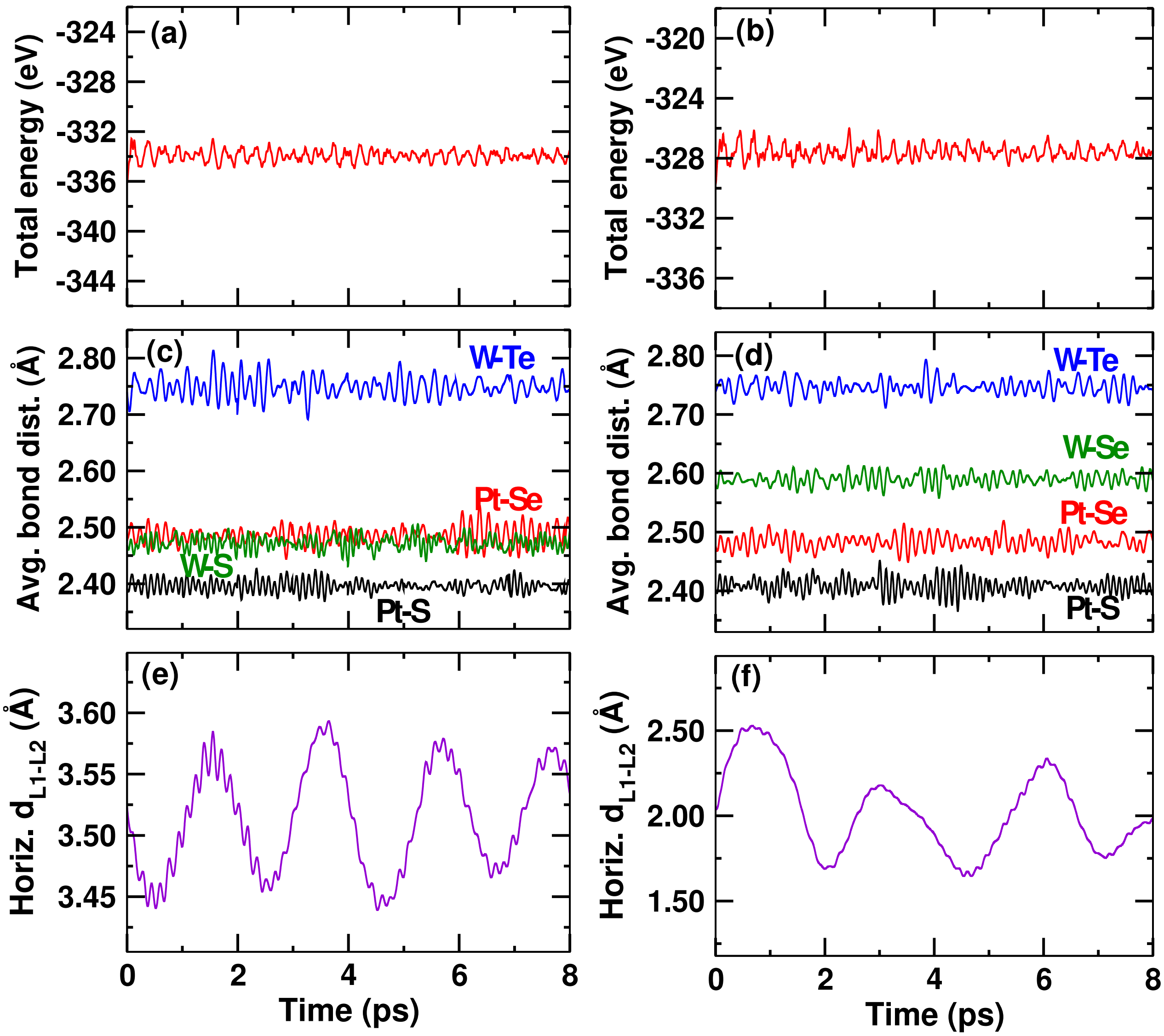

Fig. S4 Variation of total energy of the system, average bond distance (Avg. bond dist.) and horizontal distance between layers (Horizon. d_{L1-L2}) as a function of time. Left and right panels show the results for 1T-PtSSe/2H-WSTe-IC1-S3 and 1T-PtSSe/2H-WSeTe-IC2-S2 systems, respectively.

4 Band edge tuning by strain

The shift in the energy positions of the electronic bands edges for 1T-PtSSe/2H-WSeTe-IC1-S2 (-4.0 to -1.0 % strain) and 1T-PtSSe/2H-WSeTe-IC2-S2 (-1.0 and -2.0 % strain) by the application of compressive biaxial strain can be observed from the electronic band structures in Figure S8. The component layer characters for all the strained 1T-PtSSe/2H-WSeTe-IC1-S2 HSs are shown by layer-projected band structures in Figure S9. These plots suggest that strained and pristine 1T-PtSSe/2H-WSeTe-IC1-S2 has mainly type-I band alignment. The mechanism regulating the change in the band structure can be described by considering the system wavefunction as linear combination of atom-centered wavefunctions. Application of biaxial compressive or tensile strain moves the atoms closer or farther away from their equilibrium positions; this changes the overlap among atomic wavefunctions, then changing the Hamiltonian eigenvalues and the corresponding band dispersion, which determine band edge positions, band alignment and band characters. The layer projected band structures for all the strained 1T-PtSSe/2H-WSeTe-IC2-S2 are shown in Figure S10. The pristine and systems with applied compressive strains have type-II band alignment while, the systems under tensile strain show type-I band alignment.

The variation of planar averaged electrostatic potential along **c**-axis with applied tensile and compressive strains are shown in Figure S11, for 1T-PtSSe/2H-WSeTe-IC1-S2 and 1T-PtSSe/2H-WSeTe-IC2-S2. The insets of the figures show

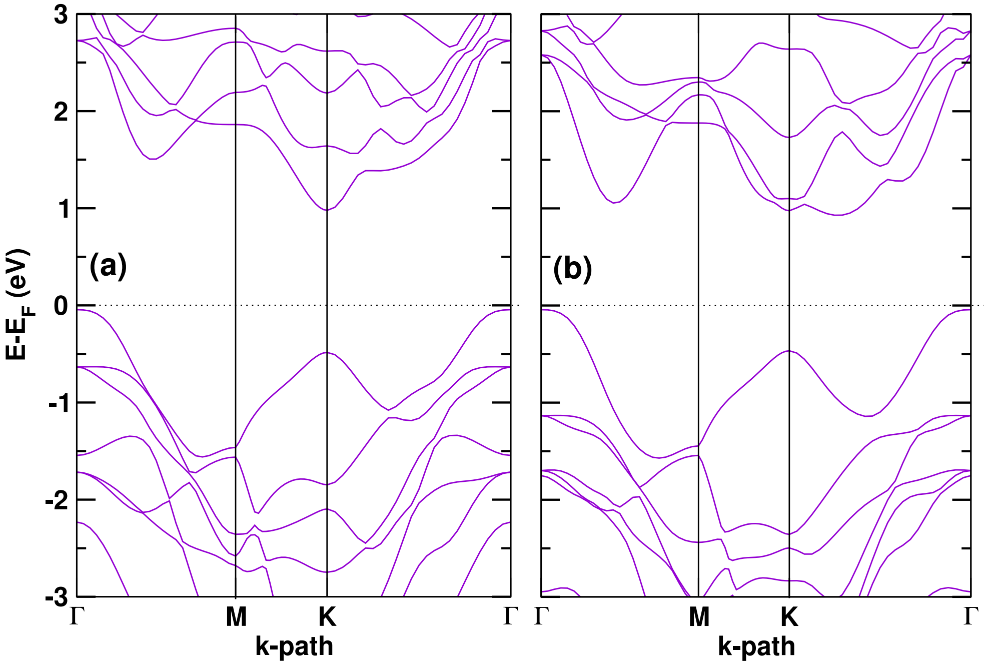

Fig. S5 Electronic band structure of (a) 1T-PtSSe/2H-WSeTe-IC1-S2 and (b) 1T-PtSSe/2H-WSeTe-IC4-S2 heterostructures calculated using the HSE06 functional.

the change in vacuum potentials at the upper (WSeTe) side and lower (PtSSe) side of the HS. Vacuum potentials at the upper and lower side of the HS are decreasing with increasing applied strain values. The photocatalytic activity of the strained 1T-PtSSe/2H-WSeTe-IC2-S2 is assessed by aligning the band edges with respect to the redox potentials; the relative energy levels of all the strained systems with the redox potentials are shown in Figure S12. By inspecting the figure, it can be observed that the valence band edges of strained 1T-PtSSe/2H-WSeTe-IC2-S2 (-1.0 and -2.0 % strains) are suitably positioned to accept electrons for the oxidation of water molecules, indicating its potential as a candidate for the OER. However, the conduction band edge positions of pristine and strained 1T-PtSSe/2H-WSeTe-IC2-S2 HSs do not suggest favourable alignment for reduction reactions.

We calculate the optical absorption spectra for strained HS candidates, 1T-PtSSe/2H-WSeTe-IC1-S2 and 1T-PtSSe/2H-WSeTe-IC2-S2 which show favourable band edge alignment for photocatalytic redox reactions. The absorption coefficient α for these systems along with pristine systems as a function of photon energy is shown in Figure S13. The spectra show absorption peaks in the visible region because of direct transitions for all the candidates. The pristine 1T-PtSSe/2H-WSeTe-IC1-S2 shows slightly higher α values in the visible region. For other systems, the application of strain does not much affect the absorption ability in the visible region.

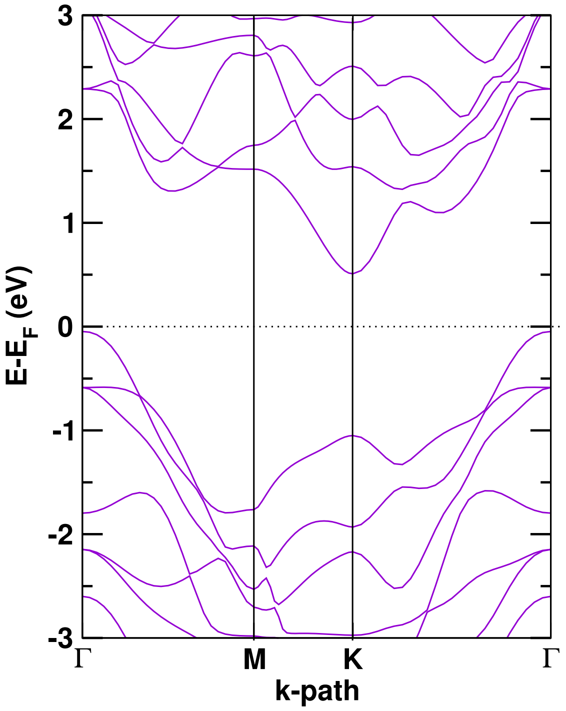

Fig. S6 Electronic band structure of 1T-PtSSe/2H-WSTe-IC1-S3 calculated using the HSE06 functional.

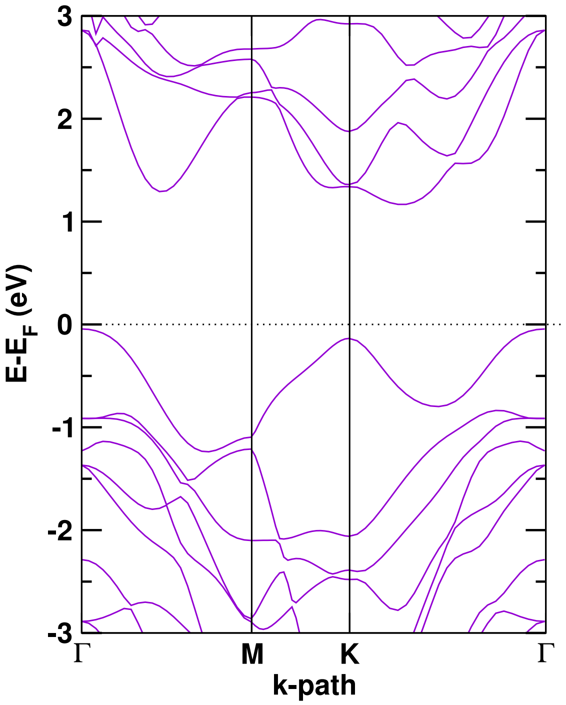

Fig. S7 Electronic band structure of 1T-PtSSe/2H-WSeTe-IC2-S2 calculated using the HSE06 functional.

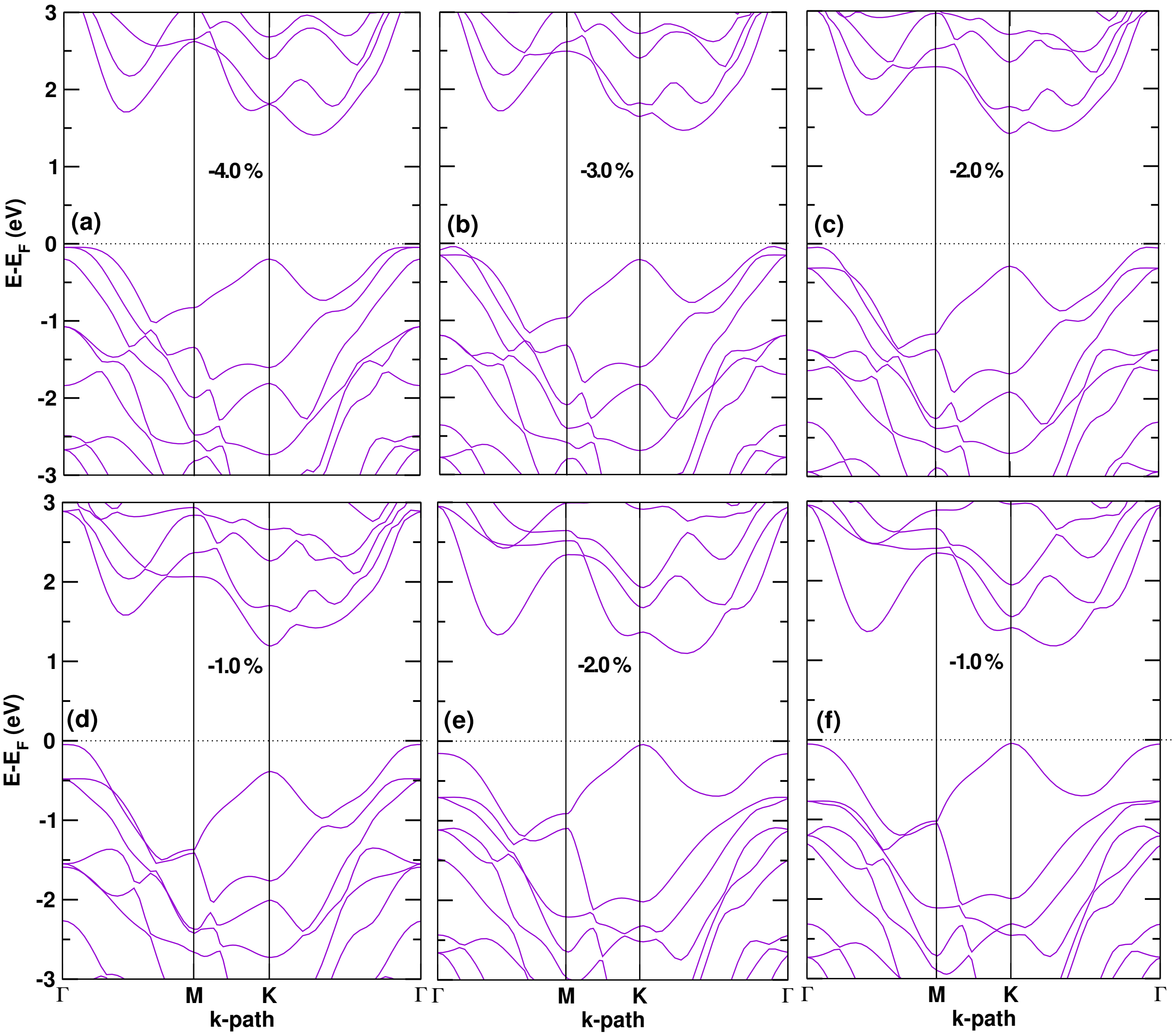

Fig. S8 Electronic band structures at different biaxial compressive strains for HS candidates for photocatalysis which show favourable band edge alignment: (a) 1T-PtSSe/2H-WSeTe-IC1-S2, -4.0 %; (b) 1T-PtSSe/2H-WSeTe-IC1-S2, -3.0 %; (c) 1T-PtSSe/2H-WSeTe-IC1-S2, -2.0 %; (d) 1T-PtSSe/2H-WSeTe-IC1-S2, -3.0 %; (e) 1T-PtSSe/2H-WSeTe-IC2-S2, -2.0 %; (e) 1T-PtSSe/2H-WSeTe-IC2-S2, -1.0 %.

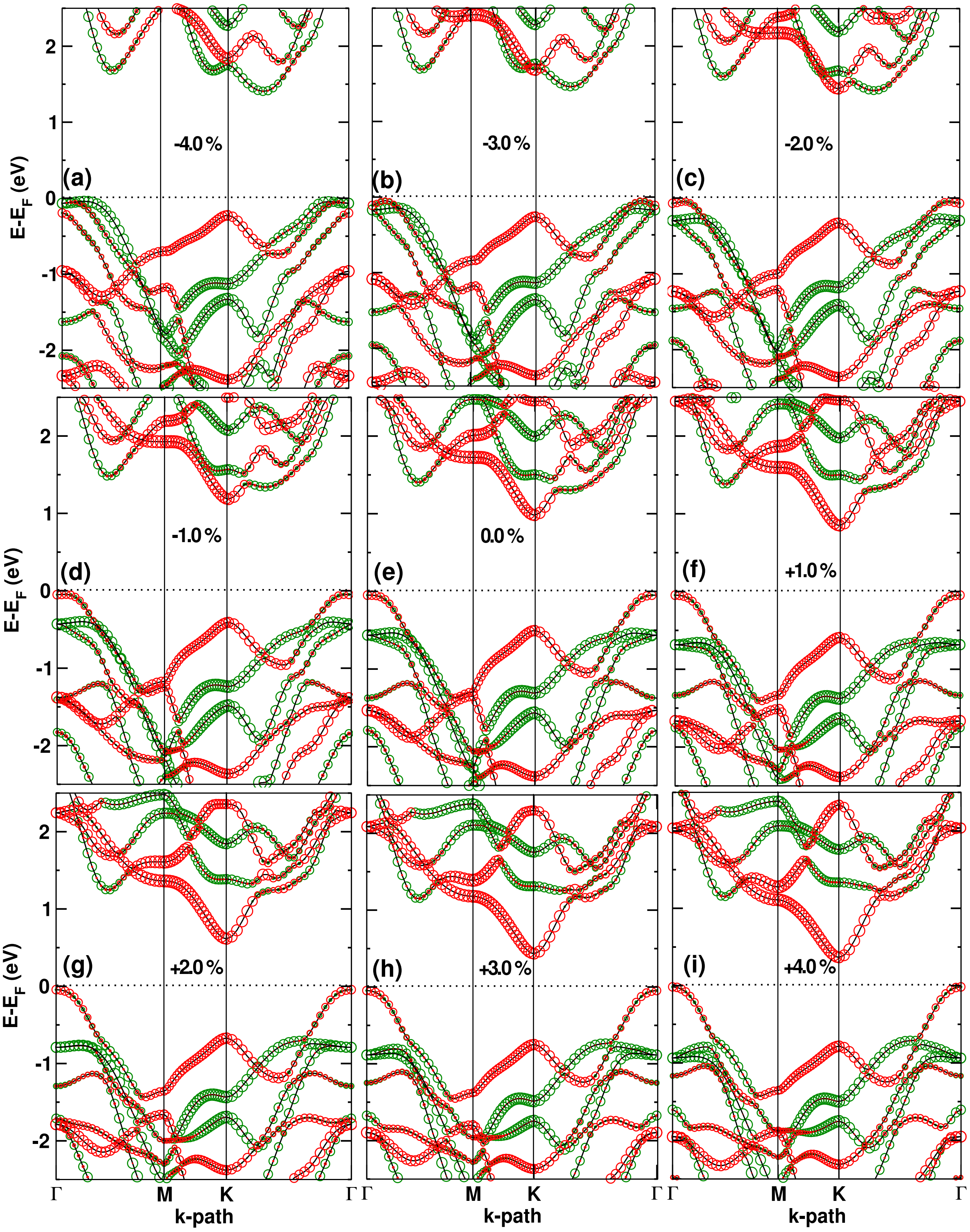

Fig. S9 Layer-projected band structures at different biaxial compressive and tensile strain values for 1T-PtSSe/2H-WSeTe-IC1-S2. Red and green circles represent projection onto WSeTe and PtSSe component monolayers, respectively, while larger circles indicate larger monolayer contributions.

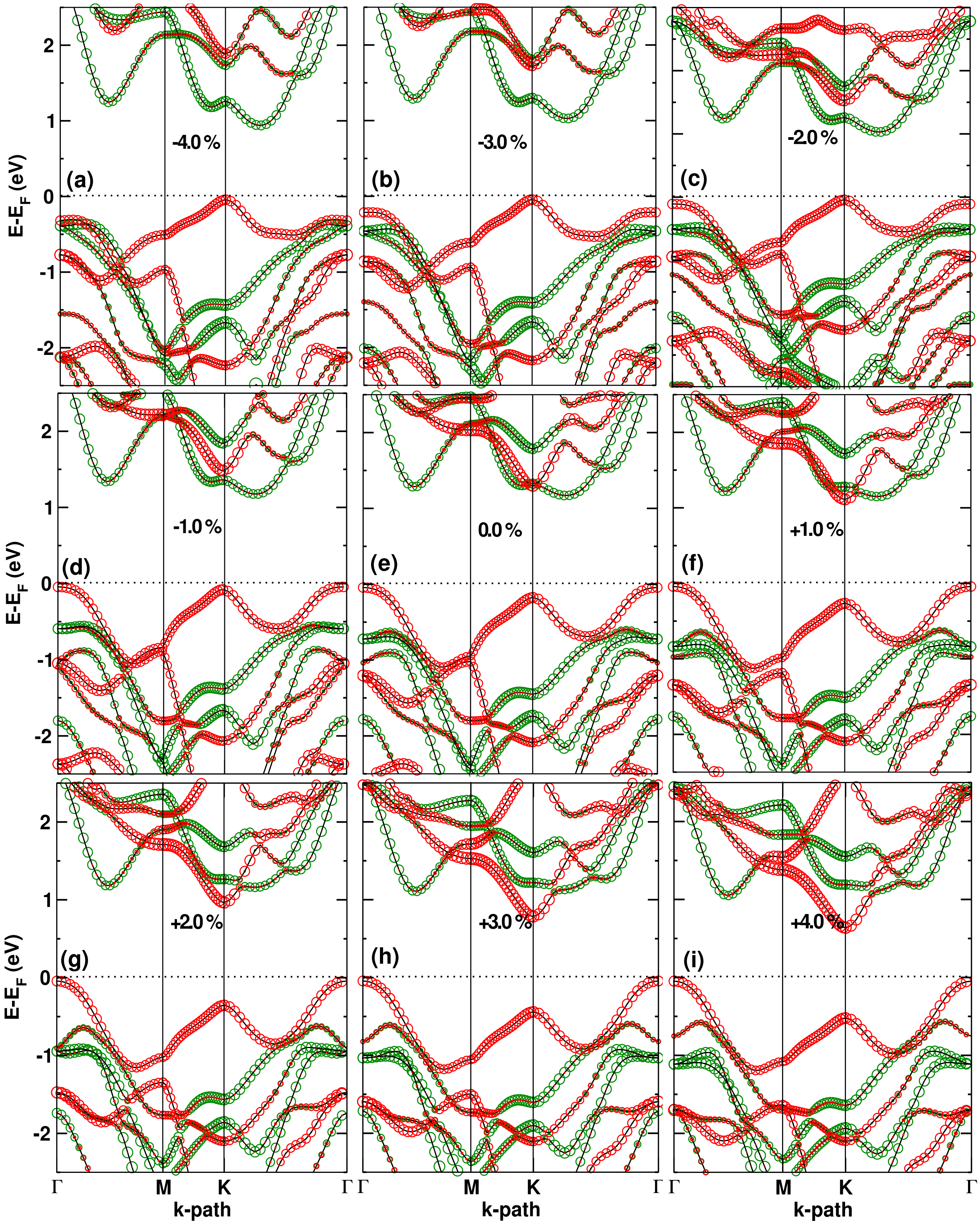

Fig. S10 Layer-projected band structures at different biaxial compressive and tensile strain values for 1T-PtSSe/2H-WSeTe-IC2-S2. Red and green circles represent projection onto WSeTe and PtSSe component monolayers, respectively, while larger circles indicate larger monolayer contributions.

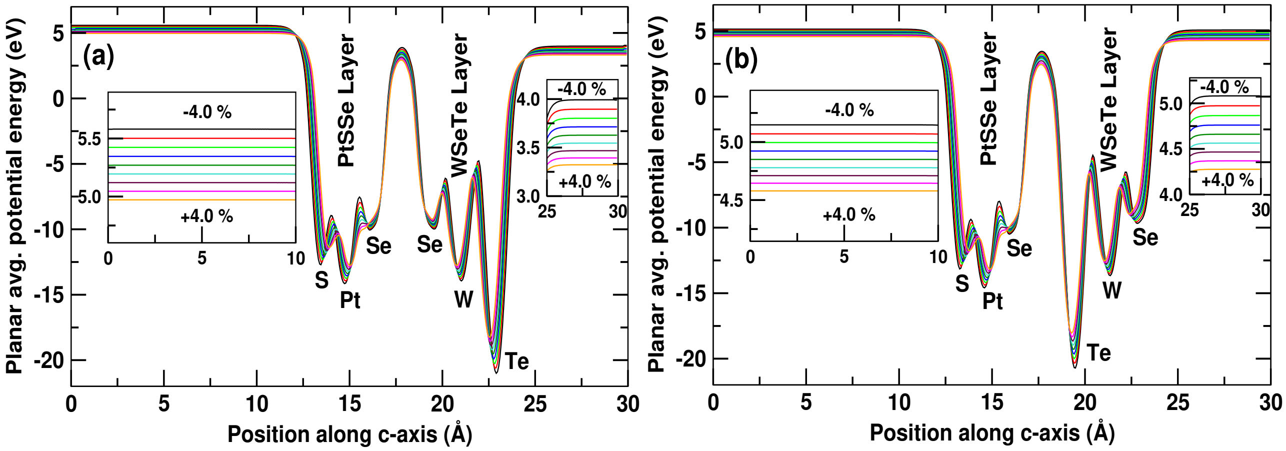

Fig. S11 Planar averaged electrostatic potential along the **c**-axis for different biaxial compressive and tensile strain values for (a) 1T-PtSSe/2H-WSeTe (PtWSeTe)-IC1-S2 and (b) 1T-PtSSe/2H-WSeTe (PtWSeTe)-IC2-S2 heterostructures. The insets on the left and right side of each figure show the magnified variation of the vacuum potential at the lower (PtSSe) side and upper (WSeTe) side. The vacuum potential is shown for the range of strain values -4.0 % to +4.0 % in order from top to bottom.

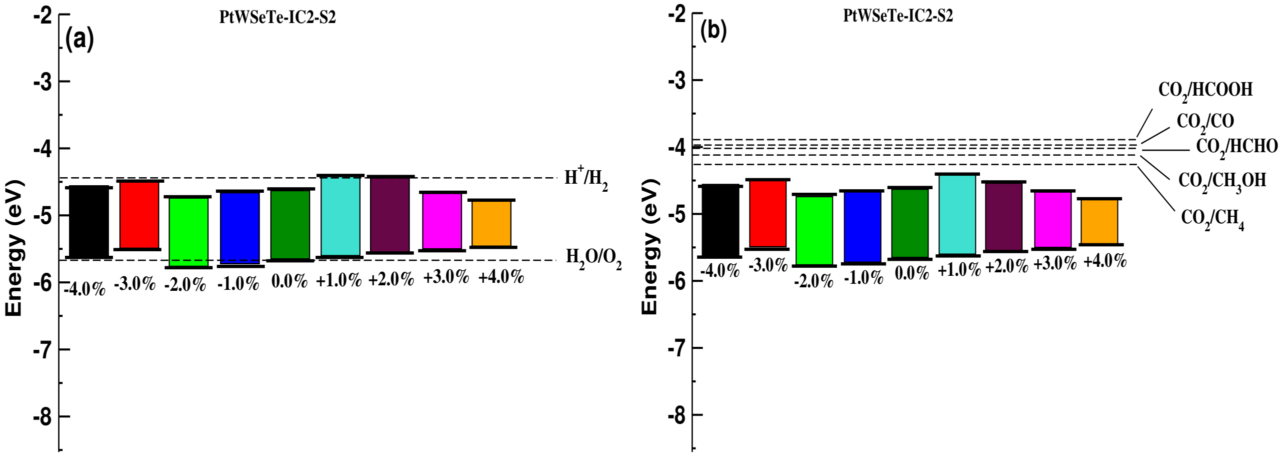

Fig. S12 Alignment of band edge positions for strained 1T-PtSSe/2H-WSeTe (PtWSeTe)-IC2-S2 heterostructures with (a) water redox potential and (b) CO_2 reduction potential.

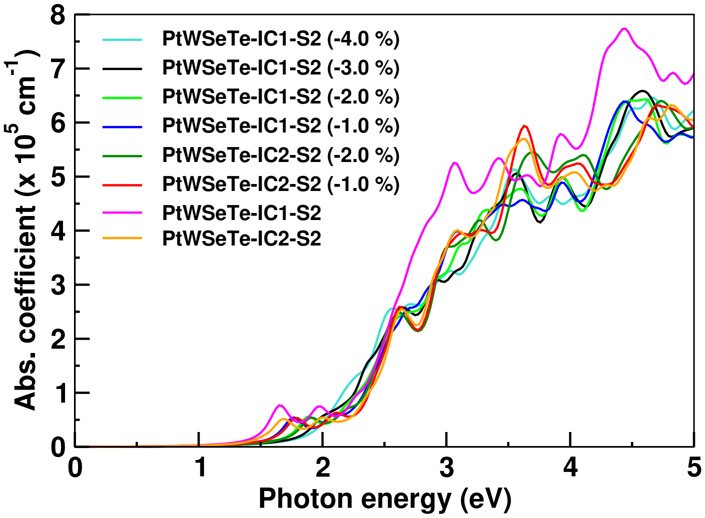

Fig. S13 Calculated optical absorption coefficient α for 1T-PtSSe/2H-WSeTe (PtWSeTe)-IC1-S2 and 1T-PtSSe/2H-WSeTe (PtWSeTe)-IC2-S2 strained heterostructures which show favourable band edge alignment for photocatalysis along with their pristine counterparts.

5 Optimized structure files

Here, we provide the geometry optimized structure files of the component monolayers and HSs of 1T-PtSSe/2H-WSSe, 1T-PtSSe/2H-WSTe and 1T-PtSSe/2H-WSeTe HSs in four atom facing types (ICs) and five stacking orders considered in POSCAR format.

5.1 1T-PtSSe monolayer

```
1T-PtSSe
   1.00000000000000
     3.6324779499665327    0.0000000000000000    0.0000000000000000
    -1.8162389749832664    3.1458181836204857    0.0000000000000000
     0.0000000000000000    0.0000000000000000   30.0000000000000000
   S    Se   Pt
     1     1     1
Direct
  0.3333333429999996  0.6666666870000029  0.4578453771697042
  0.6666666870000029  0.3333333429999996  0.5436687222117342
  0.0000000000000000  0.0000000000000000  0.4984859306185783
```

5.2 2H-WSSe monolayer

```
2H-WSSe
   1.00000000000000
     3.2607638454095018    0.0000000000000000    0.0000000000000000
    -1.6303819227047509    2.8239043255661351    0.0000000000000000
     0.0000000000000000    0.0000000000000000   30.0000000000000000
   W    Se   S
     1     1     1
Direct
  0.6666666870000029  0.3333333429999996  0.4980307788903815
  0.3333333429999996  0.6666666870000029  0.5549485235646472
  0.3333333429999996  0.6666666870000029  0.4470206975449855
```

5.3 2H-WSTe monolayer

```
2H-WSTe
   1.00000000000000
     3.3796362794363404    0.0000000000000000    0.0000000000000000
    -1.6898181397181702    2.9268508738692156    0.0000000000000000
     0.0000000000000000    0.0000000000000000   30.0000000000000000
   W    Te   S
     1     1     1
Direct
  0.6666666870000029  0.3333333429999996  0.4952511731597298
  0.3333333429999996  0.6666666870000029  0.5585934497671659
  0.3333333429999996  0.6666666870000029  0.4461553770731186
```

5.4 2H-WSeTe monolayer

```
2H-WSeTe
   1.00000000000000
```

```
     3.4539589685627483    0.0000000000000000    0.0000000000000000
    -1.7269794842813742    2.9912162104206517    0.0000000000000000
     0.0000000000000000    0.0000000000000000   30.0000000000000000
   W    Te   Se
     1     1     1
Direct
  0.6666666870000029  0.3333333429999996  0.4972823734109824
  0.3333333429999996  0.6666666870000029  0.5593110971777620
  0.3333333429999996  0.6666666870000029  0.4434065294112557

  0.00000000E+00  0.00000000E+00  0.00000000E+00
  0.00000000E+00  0.00000000E+00  0.00000000E+00
```

5.5 1T-PtSSe/2H-WSSe-IC1-S1

```
1T-PtSSe/2H-WSSe IC1-S1
   1.00000000000000
     3.4062991490817032    0.0000000000000000    0.0000000000000000
    -1.7031495745408516    2.9499415961096962    0.0000000000000000
     0.0000000000000000    0.0000000000000000   30.0000000000000000
   S    Se   Pt   W    Se   S
     1     1     1     1     1     1
Direct
  0.3333333429999996  0.6666666870000029  0.4400113977459128
  0.6666666870000029  0.3333333429999996  0.5348210427445892
  0.0000000000000000  0.0000000000000000  0.4844788826627706
  0.0000000000000000  0.0000000000000000  0.7048641662874715
  0.6666666870000029  0.3333333429999996  0.7602064451696151
  0.6666666870000029  0.3333333429999996  0.6555180963896277
```

5.6 1T-PtSSe/2H-WSSe-IC1-S2

```
1T-PtSSe/2H-WSSe IC1-S2
   1.00000000000000
     3.4121086780438374    0.0000000000000000    0.0000000000000000
    -1.7060543390219187    2.9549727957756775    0.0000000000000000
     0.0000000000000000    0.0000000000000000   30.0000000000000000
   S    Se   Pt   W    Se   S
     1     1     1     1     1     1
Direct
  0.3333333429999996  0.6666666870000029  0.4498218859112200
  0.6666666870000029  0.3333333429999996  0.5441735178287743
  0.0000000000000000  0.0000000000000000  0.4943048420909193
  0.6666666870000029  0.3333333429999996  0.6951972024737358
  0.3333333429999996  0.6666666870000029  0.7504609811066985
  0.3333333429999996  0.6666666870000029  0.6459416015886603
```

5.7 1T-PtSSe/2H-WSSe-IC1-S3

```
1T-PtSSe/2H-WSSe IC1-S3
   1.00000000000000
     3.4114423434093850    0.0000000000000000    0.0000000000000000
    -1.7057211717046925    2.9543957330537856    0.0000000000000000
```

```
      0.0000000000000000    0.0000000000000000   30.0000000000000000
    S    Se   Pt   W    Se   S
      1     1     1     1     1     1
Direct
  0.3333333429999996  0.6666666870000029  0.4506352076647246
  0.6666666870000029  0.3333333429999996  0.5450589338692637
  0.0000000000000000  0.0000000000000000  0.4950681098358061
  0.3333333429999996  0.6666666870000029  0.6943683643531884
  0.0000000000000000  0.0000000000000000  0.7496445116831865
  0.0000000000000000  0.0000000000000000  0.6451249035938389
```

5.8 1T-PtSSe/2H-WSSe-IC1-S4

```
1T-PtSSe/2H-WSSe IC1-S4
   1.00000000000000
      3.4089148106985672    0.0000000000000000    0.0000000000000000
     -1.7044574053492836    2.9522068255179410    0.0000000000000000
      0.0000000000000000    0.0000000000000000   30.0000000000000000
    S    Se   Pt   W    Se   S
      1     1     1     1     1     1
Direct
  0.3333333429999996  0.6666666870000029  0.4492951204522342
  0.6666666870000029  0.3333333429999996  0.5438577039608816
  0.0000000000000000  0.0000000000000000  0.4937802769217114
  0.6666666870000029  0.3333333429999996  0.6956588856599595
  0.0000000000000000  0.0000000000000000  0.7509542992434106
  0.0000000000000000  0.0000000000000000  0.6463537447618251
```

5.9 1T-PtSSe/2H-WSSe-IC1-S5

```
1T-PtSSe/2H-WSSe IC1-S5
   1.00000000000000
      3.4135179808941514    0.0000000000000000    0.0000000000000000
     -1.7067589904470757    2.9561932878458452    0.0000000000000000
      0.0000000000000000    0.0000000000000000   30.0000000000000000
    S    Se   Pt   W    Se   S
      1     1     1     1     1     1
Direct
  0.3333333429999996  0.6666666870000029  0.4496720044234337
  0.6666666870000029  0.3333333429999996  0.5439667045848822
  0.0000000000000000  0.0000000000000000  0.4941408840746107
  0.0000000000000000  0.0000000000000000  0.6953544828239515
  0.3333333429999996  0.6666666870000029  0.7506221024915050
  0.3333333429999996  0.6666666870000029  0.6461438526016252
```

5.10 1T-PtSSe/2H-WSSe-IC2-S1

```
1T-PtSSe/2H-WSSe IC2-S1
   1.00000000000000
      3.4060663780568809    0.0000000000000000    0.0000000000000000
     -1.7030331890284405    2.9497400104892733    0.0000000000000000
      0.0000000000000000    0.0000000000000000   30.0000000000000000
    S    Se   Pt   W    S    Se
```

```
     1     1     1     1     1     1
Direct
  0.3333333429999996  0.6666666870000029  0.4373426918871459
  0.6666666870000029  0.3333333429999996  0.5321621866941157
  0.0000000000000000  0.0000000000000000  0.4818088676420587
  0.0000000000000000  0.0000000000000000  0.7114648601037601
  0.6666666870000029  0.3333333429999996  0.7609279139728358
  0.6666666870000029  0.3333333429999996  0.6561935107000778
```

5.11 1T-PtSSe/2H-WSSe-IC2-S2

```
1T-PtSSe/2H-WSSe IC2-S2
   1.00000000000000
     3.4123609106120454    0.0000000000000000    0.0000000000000000
    -1.7061804553060227    2.9551912355875856    0.0000000000000000
     0.0000000000000000    0.0000000000000000   30.0000000000000000
   S    Se   Pt   W    S    Se
     1     1     1     1     1     1
Direct
  0.3333333429999996  0.6666666870000029  0.4470758757069362
  0.6666666870000029  0.3333333429999996  0.5414194987356993
  0.0000000000000000  0.0000000000000000  0.4915570205331932
  0.6666666870000029  0.3333333429999996  0.7018748910849055
  0.3333333429999996  0.6666666870000029  0.7512653942595264
  0.3333333429999996  0.6666666870000029  0.6467073506797121
```

5.12 1T-PtSSe/2H-WSSe-IC2-S3

```
1T-PtSSe/2H-WSSe IC2-S3
   1.00000000000000
     3.4113507138431829    0.0000000000000000    0.0000000000000000
    -1.7056753569215914    2.9543163795176013    0.0000000000000000
     0.0000000000000000    0.0000000000000000   30.0000000000000000
   S    Se   Pt   W    S    Se
     1     1     1     1     1     1
Direct
  0.3333333429999996  0.6666666870000029  0.4473607940996871
  0.6666666870000029  0.3333333429999996  0.5418166840885448
  0.0000000000000000  0.0000000000000000  0.4917771775261954
  0.3333333429999996  0.6666666870000029  0.7015620525490789
  0.0000000000000000  0.0000000000000000  0.7509721214473402
  0.0000000000000000  0.0000000000000000  0.6464112012891405
```

5.13 1T-PtSSe/2H-WSSe-IC2-S4

```
1T-PtSSe/2H-WSSe IC2-S4
   1.00000000000000
     3.4092153835998689    0.0000000000000000    0.0000000000000000
    -1.7046076917999344    2.9524671292857425    0.0000000000000000
     0.0000000000000000    0.0000000000000000   30.0000000000000000
   S    Se   Pt   W    S    Se
     1     1     1     1     1     1
Direct
```

```
  0.3333333429999996  0.6666666870000029  0.4468192918193239
  0.6666666870000029  0.3333333429999996  0.5413634643792733
  0.0000000000000000  0.0000000000000000  0.4912985656244544
  0.6666666870000029  0.3333333429999996  0.7020702204617422
  0.0000000000000000  0.0000000000000000  0.7514954561689535
  0.0000000000000000  0.0000000000000000  0.6468530325462893
```

5.14 1T-PtSSe/2H-WSSe-IC2-S5

```
1T-PtSSe/2H-WSSe IC2-S5
   1.00000000000000
     3.4125574287041780    0.0000000000000000    0.0000000000000000
    -1.7062787143520890    2.9553614252472760    0.0000000000000000
     0.0000000000000000    0.0000000000000000   30.0000000000000000
   S    Se   Pt   W    S    Se
     1     1     1     1     1     1
Direct
  0.3333333429999996  0.6666666870000029  0.4458386223338024
  0.6666666870000029  0.3333333429999996  0.5402169722193193
  0.0000000000000000  0.0000000000000000  0.4903050828857900
  0.0000000000000000  0.0000000000000000  0.7030911769843939
  0.3333333429999996  0.6666666870000029  0.7524954105425863
  0.3333333429999996  0.6666666870000029  0.6479527660340949
```

5.15 1T-PtSSe/2H-WSSe-IC3-S1

```
1T-PtSSe/2H-WSSe IC3-S1
   1.00000000000000
     3.4069369764789332    0.0000000000000000    0.0000000000000000
    -1.7034684882394666    2.9504939707793811    0.0000000000000000
     0.0000000000000000    0.0000000000000000   30.0000000000000000
   Se   S    Pt   W    S    Se
     1     1     1     1     1     1
Direct
  0.3333333429999996  0.6666666870000029  0.4375431469071955
  0.6666666870000029  0.3333333429999996  0.5322602173693980
  0.0000000000000000  0.0000000000000000  0.4879445677031669
  0.0000000000000000  0.0000000000000000  0.7093311151160719
  0.6666666870000029  0.3333333429999996  0.7587784461433742
  0.6666666870000029  0.3333333429999996  0.6540425377608088
```

5.16 1T-PtSSe/2H-WSSe-IC3-S2

```
1T-PtSSe/2H-WSSe IC3-S2
   1.00000000000000
     3.4123619301387040    0.0000000000000000    0.0000000000000000
    -1.7061809650693520    2.9551921185232488    0.0000000000000000
     0.0000000000000000    0.0000000000000000   30.0000000000000000
   Se   S    Pt   W    S    Se
     1     1     1     1     1     1
Direct
  0.3333333429999996  0.6666666870000029  0.4495592071080097
  0.6666666870000029  0.3333333429999996  0.5438698403081617
```

```
  0.0000000000000000  0.0000000000000000  0.4999652868181741
  0.6666666870000029  0.3333333429999996  0.6974330807257161
  0.3333333429999996  0.6666666870000029  0.7468105691410472
  0.3333333429999996  0.6666666870000029  0.6422620468989209
```

5.17 1T-PtSSe/2H-WSSe-IC3-S3

```
1T-PtSSe/2H-WSSe IC3-S3
   1.00000000000000
     3.4114503080753242    0.0000000000000000    0.0000000000000000
    -1.7057251540376621    2.9544026306569395    0.0000000000000000
     0.0000000000000000    0.0000000000000000   30.0000000000000000
   Se   S    Pt   W    S    Se
     1    1    1    1    1    1
Direct
  0.3333333429999996  0.6666666870000029  0.4485471415557143
  0.6666666870000029  0.3333333429999996  0.5429736143358852
  0.0000000000000000  0.0000000000000000  0.4988992559007173
  0.3333333429999996  0.6666666870000029  0.6984100043697055
  0.0000000000000000  0.0000000000000000  0.7478170006558571
  0.0000000000000000  0.0000000000000000  0.6432530141821289
```

5.18 1T-PtSSe/2H-WSSe-IC3-S4

```
1T-PtSSe/2H-WSSe IC3-S4
   1.00000000000000
     3.4086043055874606    0.0000000000000000    0.0000000000000000
    -1.7043021527937303    2.9519379202033402    0.0000000000000000
     0.0000000000000000    0.0000000000000000   30.0000000000000000
   Se   S    Pt   W    S    Se
     1    1    1    1    1    1
Direct
  0.3333333429999996  0.6666666870000029  0.4477591746389820
  0.6666666870000029  0.3333333429999996  0.5423091166265408
  0.0000000000000000  0.0000000000000000  0.4981817734192902
  0.6666666870000029  0.3333333429999996  0.6991576146253351
  0.0000000000000000  0.0000000000000000  0.7485823450063123
  0.0000000000000000  0.0000000000000000  0.6439100066835195
```

5.19 1T-PtSSe/2H-WSSe-IC3-S5

```
1T-PtSSe/2H-WSSe IC3-S5
   1.00000000000000
     3.4144084175253666    0.0000000000000000    0.0000000000000000
    -1.7072042087626833    2.9569644285885777    0.0000000000000000
     0.0000000000000000    0.0000000000000000   30.0000000000000000
   Se   S    Pt   W    S    Se
     1    1    1    1    1    1
Direct
  0.3333333429999996  0.6666666870000029  0.4486850313503297
  0.6666666870000029  0.3333333429999996  0.5429062751989093
  0.0000000000000000  0.0000000000000000  0.4990390900339392
  0.0000000000000000  0.0000000000000000  0.6983324879158417
```

```
  0.3333333429999996  0.6666666870000029  0.7477102445568988
  0.3333333429999996  0.6666666870000029  0.6432269019440753
```

5.20 1T-PtSSe/2H-WSSe-IC4-S1

```
1T-PtSSe/2H-WSSe IC4-S1
   1.00000000000000
     3.4070898385173534    0.0000000000000000    0.0000000000000000
    -1.7035449192586767    2.9506263532477264    0.0000000000000000
     0.0000000000000000    0.0000000000000000   30.0000000000000000
   Se   S    Pt   W    Se   S
     1     1     1     1     1     1
Direct
  0.3333333429999996  0.6666666870000029  0.4398426742636801
  0.6666666870000029  0.3333333429999996  0.5345564434782162
  0.0000000000000000  0.0000000000000000  0.4902463645107105
  0.0000000000000000  0.0000000000000000  0.7030977539891836
  0.6666666870000029  0.3333333429999996  0.7584226414083162
  0.6666666870000029  0.3333333429999996  0.6537341533498875
```

5.21 1T-PtSSe/2H-WSSe-IC4-S2

```
1T-PtSSe/2H-WSSe IC4-S2
   1.00000000000000
     3.4114577311239014    0.0000000000000000    0.0000000000000000
    -1.7057288655619507    2.9544090592063457    0.0000000000000000
     0.0000000000000000    0.0000000000000000   30.0000000000000000
   Se   S    Pt   W    Se   S
     1     1     1     1     1     1
Direct
  0.3333333429999996  0.6666666870000029  0.4512432935264243
  0.6666666870000029  0.3333333429999996  0.5455925479421779
  0.0000000000000000  0.0000000000000000  0.5016540461956041
  0.6666666870000029  0.3333333429999996  0.6918121583304497
  0.3333333429999996  0.6666666870000029  0.7470652875597992
  0.3333333429999996  0.6666666870000029  0.6425326974455245
```

5.22 1T-PtSSe/2H-WSSe-IC4-S3

```
1T-PtSSe/2H-WSSe IC4-S3
   1.00000000000000
     3.4110814468859192    0.0000000000000000    0.0000000000000000
    -1.7055407234429596    2.9540831874964382    0.0000000000000000
     0.0000000000000000    0.0000000000000000   30.0000000000000000
   Se   S    Pt   W    Se   S
     1     1     1     1     1     1
Direct
  0.3333333429999996  0.6666666870000029  0.4513215389491307
  0.6666666870000029  0.3333333429999996  0.5457371401465139
  0.0000000000000000  0.0000000000000000  0.5016880808291404
  0.3333333429999996  0.6666666870000029  0.6917092076065074
  0.0000000000000000  0.0000000000000000  0.7469842099787485
  0.0000000000000000  0.0000000000000000  0.6424598534899673
```

5.23 1T-PtSSe/2H-WSSe-IC4-S4

```
1T-PtSSe/2H-WSSe IC4-S4
   1.00000000000000
     3.4084401120541643    0.0000000000000000    0.0000000000000000
    -1.7042200560270822    2.9517957243980950    0.0000000000000000
     0.0000000000000000    0.0000000000000000   30.0000000000000000
   Se   S    Pt   W    Se   S
     1     1     1     1     1     1
Direct
  0.3333333429999996  0.6666666870000029  0.4495298680933786
  0.6666666870000029  0.3333333429999996  0.5440850121014691
  0.0000000000000000  0.0000000000000000  0.4999503401741876
  0.6666666870000029  0.3333333429999996  0.6934589637136028
  0.0000000000000000  0.0000000000000000  0.7487491790718650
  0.0000000000000000  0.0000000000000000  0.6441266678455762
```

5.24 1T-PtSSe/2H-WSSe-IC4-S5

```
1T-PtSSe/2H-WSSe IC4-S5
   1.00000000000000
     3.4146893946378687    0.0000000000000000    0.0000000000000000
    -1.7073446973189343    2.9572077619063082    0.0000000000000000
     0.0000000000000000    0.0000000000000000   30.0000000000000000
   Se   S    Pt   W    Se   S
     1     1     1     1     1     1
Direct
  0.3333333429999996  0.6666666870000029  0.4521738576716317
  0.6666666870000029  0.3333333429999996  0.5463395769031845
 -0.0000000000000000  0.0000000000000000  0.5025330086526540
 -0.0000000000000000  0.0000000000000000  0.6909295556871327
  0.3333333429999996  0.6666666870000029  0.7461751917300713
  0.3333333429999996  0.6666666870000029  0.6417488403553199
```

5.25 1T-PtSSe/2H-WSTe-IC1-S1

```
1T-PtSSe/2H-WSTe IC1-S1
   1.00000000000000
     3.4772818972392847    0.0000000000000000    0.0000000000000000
    -1.7386409481233638    3.0114144594162449    0.0000000000000000
     0.0000000000000000    0.0000000000000000   30.0000000000000000
   S    Se   Pt   W    Te   S
     1     1     1     1     1     1
Direct
  0.3333333429999996  0.6666666870000029  0.4425204450113185
  0.6666666870000029  0.3333333429999996  0.5342654730845595
  0.0000000000000000  0.0000000000000000  0.4857101603048761
  0.0000000000000000  0.0000000000000000  0.7021357900892298
  0.6666666870000029  0.3333333429999996  0.7642917203945387
  0.6666666870000029  0.3333333429999996  0.6542764411155204
```

5.26 1T-PtSSe/2H-WSTe-IC1-S2

```
1T-PtSSe/2h-WSTe IC1-S2
```

```
   1.00000000000000
     3.4833475634044397    0.0000000000000000    0.0000000000000000
    -1.7416737812054479    3.0166674803700588    0.0000000000000000
     0.0000000000000000    0.0000000000000000   30.0000000000000000
   S    Se   Pt   W    Te   S
     1     1     1     1     1     1
Direct
  0.3333333429999996  0.6666666870000029  0.4532804024149755
  0.6666666870000029  0.3333333429999996  0.5445658978748824
  0.0000000000000000  0.0000000000000000  0.4964806709104863
  0.6666666870000029  0.3333333429999996  0.6915366505253999
  0.3333333429999996  0.6666666870000029  0.7535699716265043
  0.3333333429999996  0.6666666870000029  0.6437664366477804
```

5.27 1T-PtSSe/2H-WSTe-IC1-S3

```
1T-PtSSe/2h-WSTe IC1-S3
   1.00000000000000
     3.4824576217890830    0.0000000000000000    0.0000000000000000
    -1.7412288103994340    3.0158967683604110    0.0000000000000000
     0.0000000000000000    0.0000000000000000   30.0000000000000000
   S    Se   Pt   W    Te   S
     1     1     1     1     1     1
Direct
  0.3333333429999996  0.6666666870000029  0.4539484070685873
  0.6666666870000029  0.3333333429999996  0.5453187177205834
  0.0000000000000000  0.0000000000000000  0.4971022069091120
  0.3333333429999996  0.6666666870000029  0.6908408068055536
  0.0000000000000000  0.0000000000000000  0.7529022111062318
  0.0000000000000000  0.0000000000000000  0.6430876803899537
```

5.28 1T-PtSSe/2H-WSTe-IC1-S4

```
1T-PtSSe/2h-WSTe IC1-S4
   1.00000000000000
     3.4794644876356431    0.0000000000000000    0.0000000000000000
    -1.7397322433215150    3.0133046381471482    0.0000000000000000
     0.0000000000000000    0.0000000000000000   30.0000000000000000
   S    Se   Pt   W    Te   S
     1     1     1     1     1     1
Direct
  0.3333333429999996  0.6666666870000029  0.4519406391306546
  0.6666666870000029  0.3333333429999996  0.5434802802980130
  0.0000000000000000  0.0000000000000000  0.4951514852546737
  0.6666666870000029  0.3333333429999996  0.6927901465964510
  0.0000000000000000  0.0000000000000000  0.7548786953850453
  0.0000000000000000  0.0000000000000000  0.6449587833351984
```

5.29 1T-PtSSe/2H-WSTe-IC1-S5

```
1T-PtSSe/2h-WSTe IC1-S5
   1.00000000000000
     3.4863995188813885    0.0000000000000000    0.0000000000000000
```

```
     -1.7431997589435149    3.0193105513823477    0.0000000000000000
      0.0000000000000000    0.0000000000000000   30.0000000000000000
   S    Se   Pt   W    Te   S
     1     1     1     1     1     1
Direct
  0.3333333429999996  0.6666666870000029  0.4542096339189783
  0.6666666870000029  0.3333333429999996  0.5453369642523356
  0.0000000000000000  0.0000000000000000  0.4973987170299239
  0.0000000000000000  0.0000000000000000  0.6906371324316254
  0.3333333429999996  0.6666666870000029  0.7526615160309404
  0.3333333429999996  0.6666666870000029  0.6429560663361968
```

5.30 1T-PtSSe/2H-WSTe-IC2-S1

```
1T-PtSSe/2h-WSTe IC2-S1
   1.00000000000000
      3.4762779463349451    0.0000000000000000    0.0000000000000000
     -1.7381389726713317    3.0105450124292519    0.0000000000000000
      0.0000000000000000    0.0000000000000000   30.0000000000000000
   S    Se   Pt   W    S    Te
     1     1     1     1     1     1
Direct
  0.3333333429999996  0.6666666870000029  0.4349783609550855
  0.6666666870000029  0.3333333429999996  0.5267713944190220
  0.0000000000000000  0.0000000000000000  0.4781776210463988
  0.0000000000000000  0.0000000000000000  0.7191389449460388
  0.6666666870000029  0.3333333429999996  0.7671407354984510
  0.6666666870000029  0.3333333429999996  0.6569929731349973
```

5.31 1T-PtSSe/2H-WSTe-IC2-S2

```
1T-PtSSe/2h-WSTe IC2-S2
   1.00000000000000
      3.4831991434342489    0.0000000000000000    0.0000000000000000
     -1.7415995712204304    3.0165389449053244    0.0000000000000000
      0.0000000000000000    0.0000000000000000   30.0000000000000000
   S    Se   Pt   W    S    Te
     1     1     1     1     1     1
Direct
  0.3333333429999996  0.6666666870000029  0.4454918352957229
  0.6666666870000029  0.3333333429999996  0.5368015062898726
 -0.0000000000000000 -0.0000000000000000  0.4887298658714790
  0.6666666870000029  0.3333333429999996  0.7087556922983305
  0.3333333429999996  0.6666666870000029  0.7566732864756478
  0.3333333429999996  0.6666666870000029  0.6467478437689833
```

5.32 1T-PtSSe/2H-WSTe-IC2-S3

```
1T-PtSSe/2h-WSTe IC2-S3
   1.00000000000000
      3.4818968629701734    0.0000000000000000    0.0000000000000000
     -1.7409484309897827    3.0154111369787300    0.0000000000000000
      0.0000000000000000    0.0000000000000000   30.0000000000000000
```

```
   S    Se   Pt   W    S    Te
     1     1     1     1     1     1
Direct
  0.3333333429999996  0.6666666870000029  0.4451048809763307
  0.6666666870000029  0.3333333429999996  0.5365687082109574
  0.0000000000000000  0.0000000000000000  0.4882305224719161
  0.3333333429999996  0.6666666870000029  0.7091065644817576
  0.0000000000000000  0.0000000000000000  0.7570632075440713
  0.0000000000000000  0.0000000000000000  0.6471261463149744
```

5.33 1T-PtSSe/2H-WSTe-IC2-S4

```
1T-PtSSe/2h-WSTe IC2-S4
   1.00000000000000
     3.4795080646657670    0.0000000000000000    0.0000000000000000
    -1.7397540318358087    3.0133423769827137    0.0000000000000000
     0.0000000000000000    0.0000000000000000   30.0000000000000000
   S    Se   Pt   W    S    Te
     1     1     1     1     1     1
Direct
  0.3333333429999996  0.6666666870000029  0.4456260285123648
  0.6666666870000029  0.3333333429999996  0.5371525754936428
  0.0000000000000000  0.0000000000000000  0.4888448875107798
  0.6666666870000029  0.3333333429999996  0.7085659489249849
  0.0000000000000000  0.0000000000000000  0.7565407985230550
  0.0000000000000000  0.0000000000000000  0.6464697910352015
```

5.34 1T-PtSSe/2H-WSTe-IC2-S5

```
1T-PtSSe/2h-WSTe IC2-S5
   1.00000000000000
     3.4825355069032131    0.0000000000000000    0.0000000000000000
    -1.7412677529548022    3.0159642188484144    0.0000000000000000
     0.0000000000000000    0.0000000000000000   30.0000000000000000
   S    Se   Pt   W    S    Te
     1     1     1     1     1     1
Direct
  0.3333333429999996  0.6666666870000029  0.4430371341384287
  0.6666666870000029  0.3333333429999996  0.5344469520433890
  0.0000000000000000  0.0000000000000000  0.4862508796405720
  0.0000000000000000  0.0000000000000000  0.7111682551437895
  0.3333333429999996  0.6666666870000029  0.7591151417206916
  0.3333333429999996  0.6666666870000029  0.6491816673132078
```

5.35 1T-PtSSe/2H-WSTe-IC3-S1

```
1T-PtSSe/2h-WSTe IC3-S1
   1.00000000000000
     3.4773139820300880    0.0000000000000000    0.0000000000000000
    -1.7386569905166434    3.0114422456641714    0.0000000000000000
     0.0000000000000000    0.0000000000000000   30.0000000000000000
   Se   S    Pt   W    S    Te
     1     1     1     1     1     1
```

```
Direct
  0.3333333429999996  0.6666666870000029  0.4358463877343342
  0.6666666870000029  0.3333333429999996  0.5275814340316172
 -0.0000000000000000  0.0000000000000000  0.4845197289728662
 -0.0000000000000000 -0.0000000000000000  0.7165312236894462
  0.6666666870000029  0.3333333429999996  0.7644121896151053
  0.6666666870000029  0.3333333429999996  0.6543090659566667
```

5.36 1T-PtSSe/2H-WSTe-IC3-S2

```
1T-PtSSe/2h-WSTe IC3-S2
   1.00000000000000
     3.4840224070527088    0.0000000000000000    0.0000000000000000
    -1.7420112030294430    3.0172519121119334    0.0000000000000000
     0.0000000000000000    0.0000000000000000   30.0000000000000000
   Se   S    Pt   W    S    Te
     1     1     1     1     1     1
Direct
  0.3333333429999996  0.6666666870000029  0.4494895844241584
  0.6666666870000029  0.3333333429999996  0.5407649089211455
  0.0000000000000000 -0.0000000000000000  0.4982023342872547
  0.6666666870000029  0.3333333429999996  0.7029723698235643
  0.3333333429999996  0.6666666870000029  0.7508236656838581
  0.3333333429999996  0.6666666870000029  0.6409471668600337
```

5.37 1T-PtSSe/2H-WSTe-IC3-S3

```
1T-PtSSe/2h-WSTe IC3-S3
   1.00000000000000
     3.4829213461749169    0.0000000000000000    0.0000000000000000
    -1.7414606725914161    3.0162983654587494    0.0000000000000000
     0.0000000000000000    0.0000000000000000   30.0000000000000000
   Se   S    Pt   W    S    Te
     1     1     1     1     1     1
Direct
  0.3333333429999996  0.6666666870000029  0.4470184496378175
  0.6666666870000029  0.3333333429999996  0.5385037520881745
 -0.0000000000000000  0.0000000000000000  0.4956377094661331
  0.3333333429999996  0.6666666870000029  0.7054465295455993
 -0.0000000000000000 -0.0000000000000000  0.7532237642420030
 -0.0000000000000000 -0.0000000000000000  0.6433698250203016
```

5.38 1T-PtSSe/2H-WSTe-IC3-S4

```
1T-PtSSe/2h-WSTe IC4-S4
   1.00000000000000
     3.4788774766185937    0.0000000000000000    0.0000000000000000
    -1.7394387378136809    3.0127962715536589    0.0000000000000000
     0.0000000000000000    0.0000000000000000   30.0000000000000000
   Se   S    Pt   W    S    Te
     1     1     1     1     1     1
Direct
  0.3333333429999996  0.6666666870000029  0.4473738191102769
```

```
  0.6666666870000029  0.3333333429999996  0.5389949350190538
  0.0000000000000000  0.0000000000000000  0.4960996888028775
  0.6666666870000029  0.3333333429999996  0.7050405280673608
 -0.0000000000000000  0.0000000000000000  0.7528740145788501
 -0.0000000000000000 -0.0000000000000000  0.6428170444216099
```

5.39 1T-PtSSe/2H-WSTe-IC3-S5

```
1T-PtSSe/2h-WSTe IC3-S5
   1.00000000000000
     3.4852304492775472    0.0000000000000000    0.0000000000000000
    -1.7426152241418515    3.0182981074031483    0.0000000000000000
     0.0000000000000000    0.0000000000000000   30.0000000000000000
   Se   S    Pt   W    S    Te
     1     1     1     1     1     1
Direct
  0.3333333429999996  0.6666666870000029  0.4466281759440420
  0.6666666870000029  0.3333333429999996  0.5378976933167934
  0.0000000000000000  0.0000000000000000  0.4952773236968255
  0.0000000000000000  0.0000000000000000  0.7058480116748171
  0.3333333429999996  0.6666666870000029  0.7536845915406190
  0.3333333429999996  0.6666666870000029  0.6438642338269176
```

5.40 1T-PtSSe/2H-WSTe-IC4-S1

```
1T-PtSSe/2h-WSTe IC4-S1
   1.00000000000000
     3.4779054209918110    0.0000000000000000    0.0000000000000000
    -1.7389527100031243    3.0119544467855177    0.0000000000000000
     0.0000000000000000    0.0000000000000000   30.0000000000000000
   Se   S    Pt   W    Te   S
     1     1     1     1     1     1
Direct
  0.3333333429999996  0.6666666870000029  0.4424856921252101
  0.6666666870000029  0.3333333429999996  0.5341417315414176
  0.0000000000000000  0.0000000000000000  0.4911316111622241
  0.0000000000000000  0.0000000000000000  0.7003951248373426
  0.6666666870000029  0.3333333429999996  0.7625292441256590
  0.6666666870000029  0.3333333429999996  0.6525166262081896
```

5.41 1T-PtSSe/2H-WSTe-IC4-S2

```
1T-PtSSe/2h-WSTe IC4-S2
   1.00000000000000
     3.4821621231468138    0.0000000000000000    0.0000000000000000
    -1.7410810610766745    3.0156408589921595    0.0000000000000000
     0.0000000000000000    0.0000000000000000   30.0000000000000000
   Se   S    Pt   W    Te   S
     1     1     1     1     1     1
Direct
  0.3333333429999996  0.6666666870000029  0.4543282024769582
  0.6666666870000029  0.3333333429999996  0.5456382430891580
  0.0000000000000000  0.0000000000000000  0.5029788235505137
```

```
  0.6666666870000029  0.3333333429999996  0.6886771211777258
  0.3333333429999996  0.6666666870000029  0.7507038424714949
  0.3333333429999996  0.6666666870000029  0.6408737972341711
```

5.42 1T-PtSSe/2H-WSTe-IC4-S3

```
1T-PtSSe/2h-WSTe IC4-S3
   1.00000000000000
     3.4817214545571811    0.0000000000000000    0.0000000000000000
    -1.7408607267860070    3.0152592288372477    0.0000000000000000
     0.0000000000000000    0.0000000000000000   30.0000000000000000
   Se   S    Pt   W    Te   S
     1     1     1     1     1     1
Direct
  0.3333333429999996  0.6666666870000029  0.4544632954207870
  0.6666666870000029  0.3333333429999996  0.5458480215196317
  0.0000000000000000  0.0000000000000000  0.5030764265372980
  0.3333333429999996  0.6666666870000029  0.6885014866547579
  0.0000000000000000  0.0000000000000000  0.7505663679230210
  0.0000000000000000  0.0000000000000000  0.6407444319444906
```

5.43 1T-PtSSe/2H-WSTe-IC4-S4

```
1T-PtSSe/2h-WSTe IC4-S4
   1.00000000000000
     3.4790919718253548    0.0000000000000000    0.0000000000000000
    -1.7395459854164070    3.0129820299919965    0.0000000000000000
     0.0000000000000000    0.0000000000000000   30.0000000000000000
   S    Se   Pt   W    S    Te
     1     1     1     1     1     1
Direct
  0.3333333429999996  0.6666666870000029  0.4521337198636459
  0.6666666870000029  0.3333333429999996  0.5436528021365987
  0.0000000000000000  0.0000000000000000  0.5007949744709634
  0.6666666870000029  0.3333333429999996  0.6907972675946752
  0.0000000000000000  0.0000000000000000  0.7528780702314322
  0.0000000000000000  0.0000000000000000  0.6429431957027560
```

5.44 1T-PtSSe/2H-WSTe-IC4-S5

```
1T-PtSSe/2h-WSTe IC4-S5
   1.00000000000000
     3.4868002123798627    0.0000000000000000    0.0000000000000000
    -1.7434001056928561    3.0196575621315414    0.0000000000000000
     0.0000000000000000    0.0000000000000000   30.0000000000000000
   Se   S    Pt   W    Te   S
     1     1     1     1     1     1
Direct
  0.3333333429999996  0.6666666870000029  0.4566697095048511
  0.6666666870000029  0.3333333429999996  0.5477069085015316
  0.0000000000000000  0.0000000000000000  0.5052738077656471
  0.0000000000000000  0.0000000000000000  0.6864011035002306
  0.3333333429999996  0.6666666870000029  0.7484023394658408
```

```
  0.3333333429999996  0.6666666870000029  0.6387461612618992
```

5.45 1T-PtSSe/2H-WSeTe-IC1-S1

```
1T-PtSSe/2H-WSeTe IC1-S1
   1.00000000000000
     3.5168703876598477    0.0000000000000000    0.0000000000000000
    -1.7584351943262397    3.0456990978764451    0.0000000000000000
     0.0000000000000000    0.0000000000000000   30.0000000000000000
   S    Se   Pt   W    Te   Se
     1     1     1     1     1     1
Direct
  0.3333333429999996  0.6666666870000029  0.4418875007299832
  0.6666666870000029  0.3333333429999996  0.5319936086147834
  0.0000000000000000  0.0000000000000000  0.4843878911537374
  0.0000000000000000  0.0000000000000000  0.7081077419176012
  0.6666666870000029  0.3333333429999996  0.7693566075967624
  0.6666666870000029  0.3333333429999996  0.6550532589871523
```

5.46 1T-PtSSe/2H-WSeTe-IC1-S2

```
1T-PtSSe/2H-WSeTe IC1-S2
   1.00000000000000
     3.5244264124533329    0.0000000000000000    0.0000000000000000
    -1.7622132067239247    3.0522428072988412    0.0000000000000000
     0.0000000000000000    0.0000000000000000   30.0000000000000000
   S    Se   Pt   W    Te   Se
     1     1     1     1     1     1
Direct
  0.3333333429999996  0.6666666870000029  0.4538373080794429
  0.6666666870000029  0.3333333429999996  0.5434247514052615
  0.0000000000000000  0.0000000000000000  0.4963357210069148
  0.6666666870000029  0.3333333429999996  0.6963326280162434
  0.3333333429999996  0.6666666870000029  0.7574448822896187
  0.3333333429999996  0.6666666870000029  0.6434113182025101
```

5.47 1T-PtSSe/2H-WSeTe-IC1-S3

```
1T-PtSSe/2H-WSeTe IC1-S3
   1.00000000000000
     3.5227995018906313    0.0000000000000000    0.0000000000000000
    -1.7613997514424606    3.0508338614226922    0.0000000000000000
     0.0000000000000000    0.0000000000000000   30.0000000000000000
   S    Se   Pt   W    Te   Se
     1     1     1     1     1     1
Direct
  0.3333333429999996  0.6666666870000029  0.4535561279676372
  0.6666666870000029  0.3333333429999996  0.5432880871346057
  0.0000000000000000  0.0000000000000000  0.4959960902790002
  0.3333333429999996  0.6666666870000029  0.6965663643290867
  0.0000000000000000  0.0000000000000000  0.7577253175838550
  0.0000000000000000  0.0000000000000000  0.6436546217058421
```

5.48 1T-PtSSe/2H-WSeTe-IC1-S4

```
1T-PtSSe/2H-WSeTe IC1-S4
   1.00000000000000
     3.5194436032417635    0.0000000000000000    0.0000000000000000
    -1.7597218021179968    3.0479275679466693    0.0000000000000000
     0.0000000000000000    0.0000000000000000   30.0000000000000000
   S    Se   Pt   W    Te   Se
     1     1     1     1     1     1
Direct
  0.3333333429999996  0.6666666870000029  0.4520073045373984
  0.6666666870000029  0.3333333429999996  0.5418958272227055
  0.0000000000000000  0.0000000000000000  0.4945261279591894
  0.6666666870000029  0.3333333429999996  0.6980604427328313
  0.0000000000000000  0.0000000000000000  0.7592480636100417
  0.0000000000000000  0.0000000000000000  0.6450488429377966
```

5.49 1T-PtSSe/2H-WSeTe-IC1-S5

```
1T-PtSSe/2H-WSeTe IC1-S5
   1.00000000000000
     3.5267972790276216    0.0000000000000000    0.0000000000000000
    -1.7633986400113248    3.0542960379809130    0.0000000000000000
     0.0000000000000000    0.0000000000000000   30.0000000000000000
   S    Se   Pt   W    Te   Se
     1     1     1     1     1     1
Direct
  0.3333333429999996  0.6666666870000029  0.4536719051750424
  0.6666666870000029  0.3333333429999996  0.5431669528554366
  0.0000000000000000  0.0000000000000000  0.4961623275542237
  0.0000000000000000  0.0000000000000000  0.6965014721581895
  0.3333333429999996  0.6666666870000029  0.7576199081302093
  0.3333333429999996  0.6666666870000029  0.6436640431269183
```

5.50 1T-PtSSe/2H-WSeTe-IC2-S1

```
1T-PtSSe/2H-WSeTe IC2-S1
   1.00000000000000
     3.5160234608445471    0.0000000000000000    0.0000000000000000
    -1.7580117309181189    3.0449656377339207    0.0000000000000000
     0.0000000000000000    0.0000000000000000   30.0000000000000000
   S    Se   Pt   W    Se   Te
     1     1     1     1     1     1
Direct
  0.3333333429999996  0.6666666870000029  0.4369923468089638
  0.6666666870000029  0.3333333429999996  0.5271406521424851
  0.0000000000000000  0.0000000000000000  0.4795035852566656
  0.0000000000000000  0.0000000000000000  0.7184123245369349
  0.6666666870000029  0.3333333429999996  0.7715640384154696
  0.6666666870000029  0.3333333429999996  0.6571736618395363
```

5.51 1T-PtSSe/2H-WSeTe-IC2-S2

```
1T-PtSSe/2H-WSeTe IC2-S2
```

```
   1.00000000000000
     3.5238223771283343    0.0000000000000000    0.0000000000000000
    -1.7619111890614558    3.0517196973622212    0.0000000000000000
     0.0000000000000000    0.0000000000000000   30.0000000000000000
   S    Se   Pt   W    Se   Te
     1     1     1     1     1     1
Direct
  0.3333333429999996  0.6666666870000029  0.4485520967204479
  0.6666666870000029  0.3333333429999996  0.5381821110632359
  0.0000000000000000  0.0000000000000000  0.4910996084978834
  0.6666666870000029  0.3333333429999996  0.7069935142795600
  0.3333333429999996  0.6666666870000029  0.7600438888593004
  0.3333333429999996  0.6666666870000029  0.6459153895795495
```

5.52 1T-PtSSe/2H-WSeTe-IC2-S3

```
1T-PtSSe/2H-WSeTe IC2-S3
   1.00000000000000
     3.5221482033980629    0.0000000000000000    0.0000000000000000
    -1.7610741021958407    3.0502698203824035    0.0000000000000000
     0.0000000000000000    0.0000000000000000   30.0000000000000000
   S    Se   Pt   W    Se   Te
     1     1     1     1     1     1
Direct
  0.3333333429999996  0.6666666870000029  0.4479030176942871
  0.6666666870000029  0.3333333429999996  0.5377082755606040
  0.0000000000000000  0.0000000000000000  0.4903358239259106
  0.3333333429999996  0.6666666870000029  0.7076025193059436
  0.0000000000000000  0.0000000000000000  0.7606964435684915
  0.0000000000000000  0.0000000000000000  0.6465405289447830
```

5.53 1T-PtSSe/2H-WSeTe-IC2-S4

```
1T-PtSSe/2H-WSeTe IC2-S4
   1.00000000000000
     3.5191079542017474    0.0000000000000000    0.0000000000000000
    -1.7595539775973770    3.0476368873512860    0.0000000000000000
     0.0000000000000000    0.0000000000000000   30.0000000000000000
   S    Se   Pt   W    Se   Te
     1     1     1     1     1     1
Direct
  0.3333333429999996  0.6666666870000029  0.4480958626194962
  0.6666666870000029  0.3333333429999996  0.5379946879299524
  0.0000000000000000  0.0000000000000000  0.4906278023209083
  0.6666666870000029  0.3333333429999996  0.7073836013234569
  0.0000000000000000  0.0000000000000000  0.7604987387287494
  0.0000000000000000  0.0000000000000000  0.6461859160773926
```

5.54 1T-PtSSe/2H-WSeTe-IC2-S5

```
1T-PtSSe/2H-WSeTe IC2-S5
   1.00000000000000
     3.5234084287755216    0.0000000000000000    0.0000000000000000
```

```
    -1.7617042148845143    3.0513612075730543    0.0000000000000000
     0.0000000000000000    0.0000000000000000   30.0000000000000000
   S    Se   Pt   W    Se   Te
     1     1     1     1     1     1
Direct
  0.3333333429999996  0.6666666870000029  0.4460967689661004
  0.6666666870000029  0.3333333429999996  0.5358117296582634
  0.0000000000000000  0.0000000000000000  0.4886227073001379
  0.0000000000000000  0.0000000000000000  0.7094068725845233
  0.3333333429999996  0.6666666870000029  0.7624905587644832
  0.3333333429999996  0.6666666870000029  0.6483579717264973
```

5.55 1T-PtSSe/2H-WSeTe-IC3-S1

```
1T-PtSSe/2H-WSeTe IC3-S1
   1.00000000000000
     3.5167443363964979    0.0000000000000000    0.0000000000000000
    -1.7583721687047076    3.0455899343298678    0.0000000000000000
     0.0000000000000000    0.0000000000000000   30.0000000000000000
   Se   S    Pt   W    Se   Te
     1     1     1     1     1     1
Direct
  0.3333333429999996  0.6666666870000029  0.4379677191164504
  0.6666666870000029  0.3333333429999996  0.5280327153948789
  0.0000000000000000  0.0000000000000000  0.4856980266198008
  0.0000000000000000  0.0000000000000000  0.7157390292883079
  0.6666666870000029  0.3333333429999996  0.7688713320254337
  0.6666666870000029  0.3333333429999996  0.6544777865551694
```

5.56 1T-PtSSe/2H-WSeTe-IC3-S2

```
1T-PtSSe/2H-WSeTe IC3-S2
   1.00000000000000
     3.5240834689815554    0.0000000000000000    0.0000000000000000
    -1.7620417349875586    3.0519458095369085    0.0000000000000000
     0.0000000000000000    0.0000000000000000   30.0000000000000000
   Se   S    Pt   W    Se   Te
     1     1     1     1     1     1
Direct
  0.3333333429999996  0.6666666870000029  0.4525006053826743
  0.6666666870000029  0.3333333429999996  0.5421234174952332
  0.0000000000000000  0.0000000000000000  0.5002799151301218
  0.6666666870000029  0.3333333429999996  0.7013093518301261
  0.3333333429999996  0.6666666870000029  0.7543382675019501
  0.3333333429999996  0.6666666870000029  0.6402350516598432
```

5.57 1T-PtSSe/2H-WSeTe-IC3-S3

```
1T-PtSSe/2H-WSeTe IC3-S3
   1.00000000000000
     3.5221510480159135    0.0000000000000000    0.0000000000000000
    -1.7610755245049079    3.0502722838939595    0.0000000000000000
     0.0000000000000000    0.0000000000000000   30.0000000000000000
```

```
   Se   S    Pt   W    Se   Te
     1     1     1     1     1     1
Direct
  0.3333333429999996  0.6666666870000029  0.4496579736698578
  0.6666666870000029  0.3333333429999996  0.5394950512029908
  0.0000000000000000  0.0000000000000000  0.4973457081308581
  0.3333333429999996  0.6666666870000029  0.7040919454664234
  0.0000000000000000  0.0000000000000000  0.7571895694482649
  0.0000000000000000  0.0000000000000000  0.6430063610816177
```

5.58 1T-PtSSe/2H-WSeTe-IC3-S4

```
1T-PtSSe/2H-WSeTe IC3-S4
   1.00000000000000
     3.5176545622536866    0.0000000000000000    0.0000000000000000
    -1.7588272816237345    3.0463782127703558    0.0000000000000000
     0.0000000000000000    0.0000000000000000   30.0000000000000000
   Se   S    Pt   W    Se   Te
     1     1     1     1     1     1
Direct
  0.3333333429999996  0.6666666870000029  0.4496486187422590
  0.6666666870000029  0.3333333429999996  0.5396407044874110
  0.0000000000000000  0.0000000000000000  0.4974382783538900
  0.6666666870000029  0.3333333429999996  0.7040665313456813
  0.0000000000000000  0.0000000000000000  0.7571881722380311
  0.0000000000000000  0.0000000000000000  0.6428043038326621
```

5.59 1T-PtSSe/2H-WSeTe-IC3-S5

```
T-PtSSe/2H-WSeTe IC3-S5
   1.00000000000000
     3.5263990467814676    0.0000000000000000    0.0000000000000000
    -1.7631995238874345    3.0539511587416772    0.0000000000000000
     0.0000000000000000    0.0000000000000000   30.0000000000000000
   Se   S    Pt   W    Se   Te
     1     1     1     1     1     1
Direct
  0.3333333429999996  0.6666666870000029  0.4503803135512214
  0.6666666870000029  0.3333333429999996  0.5399225298309247
  0.0000000000000000  0.0000000000000000  0.4981018455175814
  0.0000000000000000  0.0000000000000000  0.7034412090953381
  0.3333333429999996  0.6666666870000029  0.7564889499845506
  0.3333333429999996  0.6666666870000029  0.6424517610203893
```

5.60 1T-PtSSe/2H-WSeTe-IC4-S1

```
1T-PtSSe/2H-WSeTe IC4-S1
   1.00000000000000
     3.5174904817442765    0.0000000000000000    0.0000000000000000
    -1.7587452413685585    3.0462361151066251    0.0000000000000000
     0.0000000000000000    0.0000000000000000   30.0000000000000000
   Se   S    Pt   W    Te   Se
     1     1     1     1     1     1
```

```
Direct
  0.3333333429999996  0.6666666870000029  0.4422969687315899
  0.6666666870000029  0.3333333429999996  0.5323153825857148
  0.0000000000000000  0.0000000000000000  0.4900051405407737
  0.0000000000000000  0.0000000000000000  0.7060047868347965
  0.6666666870000029  0.3333333429999996  0.7672323947155348
  0.6666666870000029  0.3333333429999996  0.6529319355915959
```

5.61 1T-PtSSe/2H-WSeTe-IC4-S2

```
1T-PtSSe/2H-WSeTe IC4-S2
   1.00000000000000
     3.5229008011633276    0.0000000000000000    0.0000000000000000
    -1.7614504010786962    3.0509215891652004    0.0000000000000000
     0.0000000000000000    0.0000000000000000   30.0000000000000000
   Se   S    Pt   W    Te   Se
     1     1     1     1     1     1
Direct
  0.3333333429999996  0.6666666870000029  0.4555989421373354
  0.6666666870000029  0.3333333429999996  0.5452397557943556
  0.0000000000000000  0.0000000000000000  0.5033105748722022
  0.6666666870000029  0.3333333429999996  0.6928224427163343
  0.3333333429999996  0.6666666870000029  0.7539399557704272
  0.3333333429999996  0.6666666870000029  0.6398749377093225
```

5.62 1T-PtSSe/2H-WSeTe-IC4-S3

```
1T-PtSSe/2H-WSeTe IC4-S3
   1.00000000000000
     3.5220222514412258    0.0000000000000000    0.0000000000000000
    -1.7610111262177472    3.0501607427887136    0.0000000000000000
     0.0000000000000000    0.0000000000000000   30.0000000000000000
   Se   S    Pt   W    Te   Se
     1     1     1     1     1     1
Direct
  0.3333333429999996  0.6666666870000029  0.4544616481586985
  0.6666666870000029  0.3333333429999996  0.5442302666568182
  0.0000000000000000  0.0000000000000000  0.5021268015373366
  0.3333333429999996  0.6666666870000029  0.6939067932610428
  0.0000000000000000  0.0000000000000000  0.7550732450944722
  0.0000000000000000  0.0000000000000000  0.6409878542916587
```

5.63 1T-PtSSe/2H-WSeTe-IC4-S4

```
1T-PtSSe/2H-WSeTe IC4-S4
   1.00000000000000
     3.5186901570726770    0.0000000000000000    0.0000000000000000
    -1.7593450790333336    3.0472750644243627    0.0000000000000000
     0.0000000000000000    0.0000000000000000   30.0000000000000000
   Se   S    Pt   W    Te   Se
     1     1     1     1     1     1
Direct
  0.3333333429999996  0.6666666870000029  0.4527366057000179
```

```
  0.6666666870000029  0.3333333429999996  0.5426360176376548
  0.0000000000000000  0.0000000000000000  0.5004708221557621
  0.6666666870000029  0.3333333429999996  0.6955966695036935
  0.0000000000000000  0.0000000000000000  0.7567848177236201
  0.0000000000000000  0.0000000000000000  0.6425616762792075
```

5.64 1T-PtSSe/2H-WSeTe-IC4-S5

```
T-PtSSe/2H-WSeTe IC4-S5
   1.00000000000000
     3.5282438110248919    0.0000000000000000    0.0000000000000000
    -1.7641219060101276    3.0555487714378389    0.0000000000000000
     0.0000000000000000    0.0000000000000000   30.0000000000000000
   Se   S    Pt   W    Te   Se
     1     1     1     1     1     1
Direct
  0.3333333429999996  0.6666666870000029  0.4571684782695158
  0.6666666870000029  0.3333333429999996  0.5465342039773731
  0.0000000000000000  0.0000000000000000  0.5048278404805018
  0.0000000000000000  0.0000000000000000  0.6913144673562428
  0.3333333429999996  0.6666666870000029  0.7524057542065137
  0.3333333429999996  0.6666666870000029  0.6385358647098514
```
